# Teachers’ AI Belief Profiles and Their Associations with AI Use, Pedagogical Applications, and Barriers: A Person-Centered Analysis of TALIS 2024

**DOI:** 10.3390/bs16101762

**Published:** 2026-09-28

**Authors:** Jie Fang, Zhijie Jin

**Affiliations:** 1 Faculty of Education, East China Normal University, Shanghai 200062, China; 2 Berkeley School of Education, University of California, Berkeley, CA 94720, USA

**Keywords:** teachers’ AI beliefs, perceived AI utility and risk, AI use, pedagogical applications, barriers to AI use, latent profile analysis, TALIS 2024

## Abstract

Teachers’ beliefs about the utility and risks of artificial intelligence (AI) may shape AI-related practice, yet these beliefs are often treated as a single attitude and practice as simple use. This study used a person-centered approach to identify configurations of AI utility and risk beliefs and examine their associations with AI use, pedagogical applications among users, and barriers among non-users. Data were drawn from 40,680 lower-secondary teachers across 49 education systems in TALIS 2024. Following fixed-alignment assessment of approximate measurement invariance, latent profile analysis, R3STEP, and posterior-probability-weighted comparisons were conducted. Four profiles emerged: Indifferent (6.13%), Skeptical (21.41%), Measured Endorsement (56.29%), and Risk-Aware Strong Endorsement (16.18%). AI-related professional learning was most consistently associated with profile membership. Reported AI use ranged from 12.8% in the Indifferent Profile to 76.3% in the Risk-Aware Strong Endorsement Profile, which also showed the highest rates across all eight pedagogical applications. Among non-users, pedagogical reservations were most prevalent in the Indifferent and Skeptical Profiles, knowledge-and-skills constraints in the Measured Endorsement Profile, and both knowledge-and-skills and resource constraints in the Risk-Aware Strong Endorsement Profile. These findings show that distinct belief configurations correspond to meaningful heterogeneity in AI-related practice and support profile-sensitive professional learning and institutional support.

## 1. Introduction

The school classroom brings into sharp focus the central tensions that arise as artificial intelligence (AI) enters professional practice. Generative AI can assist with lesson preparation, adapt learning materials, provide individualized support, and reduce routine workload (Crompton et al., 2024; Zawacki-Richter et al., 2019), while its inaccurate outputs, bias, privacy risks, and implications for the boundaries of teachers’ professional judgment continue to attract attention (Chan, 2023; Selwyn, 2022; Williamson & Eynon, 2020). Teachers may therefore endorse the instructional value of AI and remain alert to its potential risks at the same time (Nazaretsky et al., 2022). This coexistence makes the question of how teachers organize their beliefs about the utility and risks of AI central to understanding how AI enters the instructional process.

Research on teachers and technology has developed largely within the variable-centered technology-acceptance tradition, in which perceived usefulness, ease of use, overall attitude, and behavioral intention are modeled as correlates of technology adoption (Davis, 1989; Scherer et al., 2019; Venkatesh et al., 2003). Teachers’ AI beliefs, as the term is used here, refer to teachers’ cognitive judgments of the potential instructional utility and the potential risks of AI, whereas overall attitude is a more general directional evaluation of AI; the two operate at different analytic levels. For AI, compressing utility and risk into a single favorable-to-unfavorable indicator places teachers who hold both high utility beliefs and high risk beliefs close to teachers for whom neither belief is pronounced, and thereby conceals substantively meaningful differences in belief configuration (Thompson et al., 1995). Even when utility and risk are measured separately, variable-centered models typically estimate the average association of each dimension and cannot directly reveal which combinations of utility and risk actually occur among teachers.

How teachers’ AI practice is represented is subject to a corresponding limitation. Whether or not AI is used marks only the first threshold of practice. Teachers who use AI may do so for preparatory tasks, such as working with instructional content and designing lessons. They may also use it for tasks that enter the instructional process directly, such as assessment, feedback, the analysis of learning data, and adaptive support. These two kinds of application place different demands on teachers’ professional judgment (Celik et al., 2022; Hamilton et al., 2016). Teachers who do not use AI likewise face barriers of different kinds, some reflecting constraints in the conditions for implementation and others reservations grounded in pedagogical judgment (Selwyn, 2003; Wyatt, 2003). Reducing practice to use or non-use obscures both the functional differences among users and the heterogeneity of the constraints that non-users face.

A person-centered approach offers a suitable analytic perspective for linking such belief configurations to differences in practice. It attends to the way in which several attributes jointly form patterns within the same individual, and can thereby identify latent population heterogeneity that average relationships across individuals do not reveal (Bergman & Magnusson, 1997). Existing studies have identified profiles of AI benefit and risk perceptions in the general public (Bao et al., 2022), profiles of university students’ attitudes toward ChatGPT (Y. Zhang et al., 2025), and profiles of AI use and self-efficacy among higher-education faculty (Mah & Groß, 2024). Research on school teachers has likewise identified profiles of technology integration and of technology-related beliefs (Antonietti et al., 2025; Drossel & Eickelmann, 2017; Vongkulluksn et al., 2018). What this work has not established is which configurations of perceived AI utility and perceived AI risk arise among school teachers, and how those configurations correspond to particular patterns of AI-related instructional practice. The 2024 cycle of the Teaching and Learning International Survey (TALIS), conducted by the Organisation for Economic Co-operation and Development (OECD), is the first to carry a series of items on teachers’ AI beliefs, AI use, pedagogical applications, and barriers to use (OECD, 2024, 2025). These items provide a new empirical basis for large-scale cross-system analysis. Published analyses using these data have so far remained largely variable-centered and have covered only a small number of education systems (Lei et al., 2026). Systematic evidence is therefore still lacking on the configurations of school teachers’ AI beliefs and their associations with practice.

This study draws on data from 40,680 lower-secondary teachers in 49 education systems in TALIS 2024. After assessing cross-system measurement comparability, it identifies teachers’ AI belief profiles and examines the associations of AI-related professional learning and teacher background characteristics with profile membership. It then compares AI use, the pedagogical applications reported by AI users, and the barriers faced by non-users across the profiles.

## 2. Literature Review

### 2.1. The Two-Dimensional Utility–Risk Structure of Teachers’ AI Beliefs

Research on teachers’ technology attitudes has typically represented evaluation directionally, locating it on a continuum from generally favorable to generally unfavorable. The Technology Acceptance Model and its derivatives explain technology acceptance through perceived usefulness, perceived ease of use, attitude, and behavioral intention, and the associated meta-analytic literature has largely estimated the average relationships between these constructs and technology use (Davis, 1989; Granić & Marangunić, 2019; Scherer et al., 2019). This treatment is well suited to analyzing the overall level of acceptance, but it cannot adequately represent the judgments of utility and of risk that teachers may form simultaneously when they encounter AI.

Cognitive appraisal theory provides a conceptual basis for distinguishing perceived AI utility from perceived AI risk. The theory holds that individuals appraise separately the benefits and the harms that an object or situation may bring, and that these appraisals do not necessarily move together (Lazarus & Folkman, 1984). Applied to teachers’ AI beliefs, perceived AI utility refers to the potential benefits of AI for instructional work, whereas perceived AI risk refers to its potential harms. Teachers can strongly endorse the instructional utility of AI while remaining alert to its professional, ethical, and pedagogical risks.

Research on in-service teachers provides empirical support for this two-dimensional understanding. Multi-country surveys have found that recognition of AI’s instructional potential and substantive concern about its risks frequently occur in the same teachers (Nazaretsky et al., 2022). The two appraisals also rest on different grounds: judgments of utility draw on the gains teachers expect in lesson preparation, instructional delivery, and learning support, whereas judgments of risk concern the reliability of AI output, the use of data, ethical boundaries, and the scope within which teachers’ professional judgment applies (Celik et al., 2022; Chounta et al., 2022; Selwyn, 2022). Studies that incorporate perceived risk into technology acceptance models usually position it as a constraint of roughly the same strength as perceived usefulness (Featherman & Pavlou, 2003). If the two appraisals are genuinely separable, however, the way they combine within an individual teacher is itself substantively meaningful and cannot be represented by averaging them or by allowing one to offset the other (Cacioppo & Berntson, 1994).

### 2.2. Person-Centered Approaches to Teachers’ AI Beliefs

If perceived AI utility and perceived AI risk can vary relatively independently, the key unit of description for teachers’ AI beliefs is the joint configuration of the two within the individual. Variable-centered analysis can estimate the average association of each dimension net of the other, but it cannot show which configurations actually occur among teachers, what proportion each configuration accounts for, or whether the same overall attitude score encompasses groups that differ in kind. A person-centered approach focuses on the way in which several attributes are organized within the individual and is therefore suited to these questions (Bergman & Magnusson, 1997).

Latent profile analysis (LPA) models the joint distribution of continuous indicators as a set of latent subpopulations and can identify recurring belief configurations without specifying a typology in advance (Nylund-Gibson & Choi, 2018; Spurk et al., 2020). Research on teachers and technology has demonstrated the incremental value of this perspective. Drossel and Eickelmann (2017) identified distinct profiles of teachers’ engagement with information and communication technology; Vongkulluksn et al. (2018) showed how technology value beliefs and external conditions are configured differently across teachers; and Antonietti et al. (2025) found that teachers’ technology-related beliefs and knowledge were associated with membership in technology integration profiles. This body of work reveals heterogeneity in teachers’ orientations toward technology, but it addresses educational technology in general or technology integration practices, and has not taken perceived AI utility and perceived AI risk as the core dimensions of teachers’ AI belief configurations.

Once profiles have been identified, it remains to be established which teacher characteristics are associated with membership in each profile. AI-related professional learning is of direct theoretical relevance, because it can supply technical knowledge, concrete instructional experience, and an opportunity to scrutinize algorithmic limitations and ethical issues at the same time. Research on professional learning focused on AI-based educational technology shows that explaining how AI arrives at its judgments and demonstrating how it complements teachers’ professional work can shift teachers’ trust and their willingness to use it (Nazaretsky et al., 2022). Professional learning may therefore be associated with greater endorsement of utility, and it may equally be accompanied by a fuller recognition of risk. Background characteristics such as age, teaching experience, and educational attainment reflect different professional trajectories and knowledge bases, but existing cross-national evidence has not shown a consistent direction of association between these characteristics and teachers’ AI beliefs (Viberg et al., 2025). Professional learning and background characteristics are therefore appropriate correlates of profile membership to examine, with the direction of their associations left to the data.

### 2.3. Differentiation in Practice Across Teachers’ AI Belief Profiles

The value of studying teachers’ AI belief profiles also depends on whether the different configurations are accompanied by observable differences in practice. The literature suggests that AI practice involves at least three connected but non-interchangeable levels: whether teachers use AI, the instructional functions for which users apply AI, and the barriers that non-users face. Distinguishing these three levels makes it possible to show how the association between belief configurations and practice extends from use status to specific instructional functions and implementation conditions.

#### 2.3.1. AI Use: The Behavioral Correlates of Belief Configurations

Research on teachers and technology has consistently found that more favorable beliefs about the value of technology are associated with higher levels of technology use, even though access, training, and institutional conditions remain important (Ertmer, 2005; Scherer et al., 2019). Work on AI has likewise linked teachers’ perceived utility, trust, and preparedness to their willingness to use AI or to actual use (Granström & Oppi, 2025; Nazaretsky et al., 2022). On this basis, profiles with higher perceived AI utility would be expected to show higher rates of AI use.

Risk beliefs complicate this relationship. Higher perceived risk may restrain use, but it may equally reflect the considered judgment that teachers form once they have actually engaged with AI. For teachers who hold high utility beliefs and high risk beliefs at the same time in particular, a single overall attitude yields no clear prediction. Comparing AI use rates across belief profiles makes it possible to test whether different within-person combinations of utility and risk correspond to different rates of reported use, and to establish whether alertness to risk is necessarily accompanied by less use.

#### 2.3.2. Pedagogical Applications: Functional Differentiation in AI Practice

A binary indicator of AI use cannot reveal where AI is used within instructional work. Research on classroom technology distinguishes applications that mainly substitute for or supplement existing practice from applications that are more directly involved in instructional decision-making and the learning process (Hamilton et al., 2016). For AI, tasks such as working with instructional content and designing lessons and activities primarily support instructional preparation, whereas tasks such as assessment, feedback and communication, the analysis of learning data, the adjustment of learning materials, and support for students with special learning needs bear more directly on the process of student learning (Celik et al., 2022; C. Zhang et al., 2026). These functions serve different roles in instructional preparation, instructional decision-making, and the support of student learning, and they place different demands on teachers’ contextual judgment, verification responsibilities, and professional oversight.

Teachers’ belief configurations may be associated with the range of instructional functions for which they use AI. Higher utility beliefs may support broader application, whereas higher risk beliefs may make teachers more cautious in tasks with more immediate professional consequences, such as assessment, feedback, and adaptive support. Existing research has focused largely on whether teachers use AI or on the overall frequency of use, and has paid limited attention to functional differences among users. Comparing profile-specific proportions across the individual pedagogical applications makes it possible to test further whether belief heterogeneity extends to the functional composition of AI practice.

#### 2.3.3. Barriers to AI Use: Heterogeneous Constraints Among Non-Users

The implementation conditions faced by teachers who report not using AI are markedly heterogeneous. Research on technology use has long distinguished several kinds of barrier: knowledge and skills, resources and infrastructure, institutional arrangements, and value and pedagogical beliefs (Ertmer, 1999; Selwyn, 2003; Wyatt, 2003). Studies of AI in educational settings have likewise found that teachers may face insufficient knowledge and skills (Al-Abdullatif, 2024; Kohnke et al., 2023; Shahzad et al., 2025), resource and infrastructure constraints or school regulations (Chatterjee & Bhattacharjee, 2020; Moorhouse et al., 2024), and may hold reservations about the pedagogical appropriateness of AI (Gokturk-Saglam, 2026). The support that these barriers call for differs markedly. Such constraints may be associated with teachers’ beliefs: teachers who strongly endorse the utility of AI may nonetheless be limited by knowledge, skills, or resource conditions, whereas teachers with weaker endorsement of utility or heightened alertness to risk may more often report reservations about the pedagogical appropriateness of AI. The barriers reported by non-users therefore provide a largely unexplored basis for examining the association between belief configurations and implementation conditions.

## 3. Research Framework and Research Questions

Synthesizing the preceding theoretical arguments and empirical evidence, this study takes the within-person configuration of perceived AI utility and perceived AI risk as its analytic core. The design proceeds from identifying latent profiles, through examining correlates of profile membership, to comparing differences in AI practice across profiles. Figure 1 translates these conceptual relations into five analytic tasks. The corresponding research questions are as follows.

RQ1. What latent profiles of teachers’ AI beliefs can be identified among lower-secondary teachers from 49 education systems?
RQ2. How are AI-related professional learning and teacher background characteristics associated with AI belief profile membership?
RQ3. How does AI use differ across teachers’ AI belief profiles?
RQ4. Among teachers who reported using AI, how do pedagogical applications of AI differ across AI belief profiles?
RQ5. Among teachers who reported no AI use, how do reported barriers to AI use differ across AI belief profiles?

## 4. Method

### 4.1. Data and Sample

This study used cross-sectional teacher-level data from the TALIS 2024 sample at level 2 of the International Standard Classification of Education (ISCED; OECD, 2025).

The analytic sample was constructed in three steps. First, of the 194,521 teachers in the TALIS 2024 ISCED level 2 sample, 64,904 were assigned to a questionnaire form containing the AI belief items. TALIS special missing-value codes and out-of-range values were recoded as missing in accordance with the official coding rules, leaving 47,984 teachers in the initial valid sample. Second, to retain sufficient information for the multi-item measures used in the cross-system analyses, and informed by the way TALIS and the OECD treat multi-item constructs, teachers were required to have at least two valid responses on each AI belief dimension and valid data on AI-related professional learning and teacher background variables. This yielded a main analytic sample of 40,680 teachers from 49 education systems; 22,769 teachers with valid responses to all ten AI belief items formed the complete-response subsample. Third, based on reported AI use (TT4G36), the main sample was divided into 20,394 AI users and 20,286 non-users. The user subsample was used to compare pedagogical applications across AI belief profiles, whereas the non-user subsample was used to compare barriers to AI use across profiles. Figure 2 presents the sample selection process, and Table 1 reports the characteristics of the main analytic sample.

### 4.2. Research Instruments

#### 4.2.1. Teachers’ AI Beliefs

Teachers’ AI beliefs were measured using ten items from the TALIS 2024 teacher questionnaire (TT4G35A–TT4G35J; OECD, 2024). The first five items captured perceived AI utility, reflecting teachers’ judgments of AI’s potential contributions to instructional design, adaptation of learning materials, individualized support, support for students with special learning needs, and reduction in routine workload. The remaining five items captured perceived AI risk, reflecting teachers’ judgments of the potential harms of AI use with respect to authorship of student work, information reliability, bias and misconceptions, data protection, and the appropriateness of instructional suggestions. Before the TT4G35 items, TALIS 2024 defined artificial intelligence as the capacity of computers to carry out tasks that have conventionally been regarded as requiring human intelligence, giving prediction, decision support, and text generation as examples (OECD, 2024). Working from this function-based definition, the present study used the TT4G35 items to characterize teachers’ overall appraisals of the utility and risks of AI and to identify their AI belief profiles. Items were rated on a four-point agreement scale from 1 (strongly disagree) to 4 (strongly agree), with an additional option, 5 (I do not know). Because “I do not know” does not indicate a definite level of agreement and may reflect an unformed judgment or insufficient knowledge (Krosnick, 1991), it was coded as missing; the remaining four response categories were treated as ordered categorical variables.

The two-factor structure of perceived AI utility and perceived AI risk was evaluated using the weighted least squares mean- and variance-adjusted estimator (WLSMV), with teacher weights rescaled within education systems and standard errors adjusted for school-level clustering. The model fit the data well, χ^2^(34) = 5721.068, *p* < 0.001, CFI = 0.984, TLI = 0.979, RMSEA = 0.059, SRMR = 0.043. Standardized factor loadings ranged from 0.736 to 0.939 for perceived AI utility (Cronbach’s α = 0.911, composite reliability [CR] = 0.936, average variance extracted [AVE] = 0.746) and from 0.667 to 0.860 for perceived AI risk (α = 0.837, CR = 0.874, AVE = 0.583).

#### 4.2.2. AI Use, Pedagogical Applications, and Barriers to AI Use

Teachers’ AI use was measured with TT4G36, which asked whether teachers had used AI tools during the previous 12 months. Responses were coded 1 for reported use and 0 for non-use.

For teachers who reported using AI, TT4G37A–TT4G37H measured eight pedagogical applications: supporting assessment, working with instructional content, designing lessons and activities, supporting students with special learning needs, adjusting material difficulty, generating feedback and communication texts, analyzing student engagement and performance, and skill practice in authentic contexts. Each application was coded 1 when reported and 0 otherwise. TT4G37I, which covered other uses, was excluded from the core analyses because responses could not be interpreted on a common substantive basis.

For teachers who reported no AI use, TT4G38A–TT4G38E measured five barriers to AI use: resource and infrastructure constraints, insufficient knowledge and skills, reservations about the pedagogical appropriateness of AI, school-level restrictions on AI use, and the burden of integrating new technology. Each barrier was coded 1 when reported and 0 otherwise. TT4G38F, which covered other reasons, was excluded from the core analyses for the same reason.

#### 4.2.3. AI Professional Learning and Teacher Background Characteristics

AI professional learning was measured with TT4G21G, which asked whether teachers had participated in professional learning activities related to using AI for teaching and learning during the previous 12 months. The variable was coded 1 for participation and 0 for non-participation. Teacher background characteristics comprised age, teaching experience, and highest educational attainment. Where TALIS provides official derived indicators, the official coding was retained to ensure consistent definitions across education systems.

### 4.3. Analytic Strategy

Analyses were conducted in Mplus 8.3 (Muthén & Muthén, Los Angeles, CA, USA) and Stata 18 (StataCorp LLC, College Station, TX, USA). Mplus was used for measurement invariance testing, LPA, R3STEP for covariate analysis, and Wald tests; Stata was used for posterior-probability-weighted estimation and cross-system analyses. For pooled analyses, teacher sampling weights (TCHWGT) were rescaled within each education system to sum to 1, so that each of the 49 systems contributed equally to the pooled estimates.

To address RQ1, a configural multigroup confirmatory factor analysis (MGCFA) was first estimated across the 49 education systems with WLSMV, followed by fixed alignment using robust maximum likelihood estimation to assess the approximate measurement invariance of teachers’ AI beliefs and to obtain factor scores for perceived AI utility and perceived AI risk (Asparouhov & Muthén, 2014b). LPA models with two to six profiles were then estimated using these factor scores, and the final model was selected on the basis of the log-likelihood, the Akaike information criterion (AIC), the Bayesian information criterion (BIC), the sample-size adjusted BIC (SSA-BIC), entropy, the Lo–Mendell–Rubin likelihood ratio test, profile size, and substantive interpretability (Nylund-Gibson & Choi, 2018; Peugh & Fan, 2013). The retained profiles were named according to their score patterns on the two belief dimensions. Profile-specific means were compared using Wald tests, with Holm adjustment applied to pairwise comparisons. Spain served as the reference system in the main fixed alignment analysis, and Kazakhstan was used as an alternative reference in a sensitivity analysis. The LPA was also repeated in the complete-response subsample to assess the sensitivity of profile identification to the sample inclusion criteria.

To address RQ2, the R3STEP procedure was used to examine the associations of AI professional learning and teacher background characteristics with AI belief profile membership while correcting for classification error (Asparouhov & Muthén, 2014a; Vermunt, 2010). Education-system fixed effects were included, and standard errors were adjusted for school-level clustering.

To address RQ3–RQ5, profile-specific proportions were estimated using posterior class probabilities jointly with teacher weights rescaled within education systems. Taylor linearization accounting for school-level clustering was used to estimate 95% confidence intervals (CIs) and conduct Wald F tests. RQ3 compared AI use rates across profiles; RQ4 compared the eight pedagogical applications among AI users; and RQ5 compared the five barriers to AI use among non-users.

Cross-system stability was further examined by comparing AI belief profile characteristics across the 49 education systems and by estimating logistic associations of perceived AI utility and perceived AI risk with AI use, both in the pooled sample and separately within each system. System-specific estimates were then pooled using restricted maximum likelihood (REML) random-effects models to assess the cross-system heterogeneity of these associations.

## 5. Results

### 5.1. Cross-System Comparability of Teachers’ AI Beliefs

The configural MGCFA across the 49 education systems showed acceptable fit, χ^2^(1666) = 13,504.951, *p* < 0.001, CFI = 0.975, TLI = 0.967, RMSEA = 0.085, SRMR = 0.063. Fixed alignment identified 122 of the 980 intercept and loading parameters (12.4%) as non-invariant. The corresponding proportion for loading parameters was 3.5%, indicating that item loadings were largely stable across systems. Under the alternative reference system, correlations between factor scores from the two specifications were 0.9999 for perceived AI utility and 0.9977 for perceived AI risk, indicating high stability of factor scoring across reference-system choices. Supplementary Table S1 reports the parameter-level diagnostics and the reference-system sensitivity results.

### 5.2. Identification and Characteristics of Teachers’ AI Belief Profiles (RQ1)

LPA models were estimated using the factor scores for the two AI belief dimensions. Table 2 reports the fit results for the two- to six-profile models. AIC, BIC, and SSA-BIC decreased continuously as the number of profiles increased, with the largest reduction in BIC occurring from the three- to the four-profile model (ΔBIC = 10,814.724) and smaller reductions thereafter. Although the five- and six-profile models yielded lower information criteria, both produced profiles with small proportions, and the additional profiles did not form substantively meaningful belief configurations. Taking classification quality, profile size, and interpretability together, the four-profile model was retained (entropy = 0.941). When the fixed alignment reference system was changed, profile classification agreement was 99.62%, with an adjusted Rand index of 0.989. Repeating the LPA in the complete-response subsample also yielded essentially the same profile structure (Supplementary Table S2), indicating that the four-profile solution was highly stable across both reference-system choice and sample inclusion criteria.

Figure 3 shows the levels of perceived AI utility and perceived AI risk for the four profiles relative to the overall weighted mean across the 49 education systems, and Table 3 reports the corresponding Wald tests, Holm-adjusted pairwise comparisons, and effect sizes. C1 scored far below the overall weighted mean on perceived AI utility and slightly below it on perceived AI risk, and was labeled the Indifferent Profile (6.13%). C2 scored below the overall weighted mean on perceived AI utility and slightly above it on perceived AI risk, and was labeled the Skeptical Profile (21.41%). C3 scored above the overall weighted mean on perceived AI utility and close to it on perceived AI risk, and was labeled the Measured Endorsement Profile (56.29%). C4 had the highest perceived AI utility score, while its perceived AI risk score was slightly above the overall weighted mean and the highest among the four profiles; it was labeled the Risk-Aware Strong Endorsement Profile (16.18%). For perceived AI utility, the four profiles formed a clear gradient, C4 > C3 > C2 > C1 (Wald χ^2^(3) = 164,209.924, *p* < 0.001). Perceived AI risk also differed across profiles (Wald χ^2^(3) = 106.178, *p* < 0.001), with the ordering C4 > C2 > C3 > C1, but the differences between profiles were considerably smaller. Effect sizes for the pairwise comparisons ranged from Cohen’s |d| = 4.123 to 13.416 for perceived AI utility and from 0.056 to 0.335 for perceived AI risk.

The relative ordering of the four profiles on perceived AI utility was consistent across all 49 education systems, whereas their ordering on perceived AI risk varied to some extent. Profile proportions and profile characteristics on the two belief dimensions for each education system are reported in Supplementary Tables S3 and S4. Overall, teachers’ AI beliefs were not simply distributed along a single continuum from support to opposition; rather, they formed distinct combinations of perceived utility and perceived risk. The ordering of the profiles on perceived AI risk was not monotonic in perceived AI utility. C2, for example, scored lower than C3 on perceived AI utility but higher on perceived AI risk. Between-profile differentiation was carried mainly by perceived AI utility, whereas perceived AI risk provided a smaller additional differentiation.

### 5.3. Associations of AI Professional Learning and Teacher Background Characteristics with AI Belief Profile Membership (RQ2)

Table 4 reports the results of the R3STEP multinomial logistic regression. C1 served as the reference profile, and the model included AI professional learning, teacher background characteristics, and education-system fixed effects. Relative to teachers who had not participated in AI professional learning, those who had participated had higher odds of membership in C2, C3, and C4 rather than C1, with odds ratios (ORs) of 1.607, 3.113, and 5.856, respectively (all *p* < 0.001); the ORs increased successively from C2 to C4. Age was not significant in any of the three profile contrasts. Relative to teachers with five years or less of teaching experience, those with 6–10 and 11–20 years had lower odds of membership in C3 rather than C1 (OR = 0.767 and 0.770) and lower odds of membership in C4 rather than C1 (OR = 0.755 and 0.615); for teachers with more than 20 years of experience, the association was significant only in the C4 versus C1 contrast (OR = 0.626). Significant associations for educational attainment were likewise confined to a small number of contrasts: teachers with upper secondary education or below had lower odds of membership in C2 rather than C1 (OR = 0.439), and teachers with a master’s or doctoral degree had lower odds of membership in C3 rather than C1 (OR = 0.810 and 0.546).

Overall, AI professional learning showed a more systematic pattern of association with profile membership, whereas associations for teacher background characteristics were more limited.

### 5.4. Differences in AI Use Across AI Belief Profiles (RQ3)

Figure 4 shows AI use rates by AI belief profile. Using teacher weights rescaled so that each education system contributed equally to the estimate, 49.5% of teachers reported using AI for teaching or for supporting student learning during the previous 12 months. AI use rates differed significantly across the four profiles and followed the gradient C4 > C3 > C2 > C1, *F*(3, 10,199) = 1436.459, *p* < 0.001. The rate was highest in C4 (76.3%), followed by C3 (54.8%), C2 (25.9%), and C1 (12.8%).

As a supplementary cross-system analysis, the cross-system consistency of the associations of perceived AI utility and perceived AI risk with AI use was examined. In the pooled sample, perceived AI utility was positively associated with AI use (OR = 2.740, 95% CI [2.611, 2.874], *p* < 0.001), whereas perceived AI risk was negatively associated with it (OR = 0.820, 95% CI [0.788, 0.853], *p* < 0.001). A product term for the two dimensions was then added to the pooled model. The interaction was negative and significant (b = −0.050, SE = 0.020, OR = 0.952, 95% CI [0.914, 0.990], *p* = 0.015): the negative association of perceived AI risk with AI use was stronger at higher levels of perceived AI utility. The main-effects model was also estimated separately within each of the 49 education systems. Perceived AI utility was significantly and positively associated with AI use in all 49 systems; the REML random-effects summary OR was 2.866 (95% CI [2.604, 3.155], I^2^ = 80.08%). Perceived AI risk was negatively associated with AI use in 40 systems and positively associated in nine; the summary OR was 0.844 (95% CI [0.800, 0.889], I^2^ = 52.07%). The pooled models and the random-effects summary are reported in Supplementary Table S5; the system-specific estimates are reported in Supplementary Table S6 and the corresponding forest plot in Supplementary Figure S1.

### 5.5. Differences in Pedagogical Applications Across AI Belief Profiles Among AI Users (RQ4)

Among teachers who reported using AI, the most commonly reported applications were working with instructional content (73.9%) and designing lessons and activities (72.6%), followed by skill practice in authentic contexts (54.0%) and adjusting material difficulty (49.7%). Supporting students with special learning needs (43.9%) and generating feedback and communication texts (43.6%) were reported at intermediate rates, whereas analyzing student engagement and performance (36.3%) and supporting assessment (34.4%) were the least commonly reported. Overall, applications were more commonly reported for content preparation and instructional design than for assessment and the analysis of learning data. Profile-specific proportions and 95% CIs for the eight pedagogical applications are reported in Supplementary Table S7.

Significant between-profile differences were observed for all eight pedagogical applications (all *p* < 0.001). As Figure 5 shows, the Risk-Aware Strong Endorsement Profile (C4) had the highest reported proportion for each of the eight applications, most markedly for designing lessons and activities (83.4%), working with instructional content (82.7%), and adjusting material difficulty (66.1%); the Measured Endorsement Profile (C3) ranked second across applications. Proportions were generally lower in the Indifferent Profile (C1) and the Skeptical Profile (C2), but their application patterns were not identical: C1 showed higher proportions than C2 for supporting assessment, supporting students with special learning needs, adjusting material difficulty, generating feedback and communication texts, and analyzing student engagement and performance, whereas C2 showed higher proportions than C1 for working with instructional content and designing lessons and activities. Across profiles, reported use was concentrated more heavily in preparatory tasks related to content development and instructional design, whereas assessment- and learning-data-related uses were less common.

### 5.6. Differences in Barriers to AI Use Across AI Belief Profiles Among Non-Users (RQ5)

Among teachers who reported no AI use, the most common barrier was insufficient knowledge and skills (64.7%), followed by reservations about the pedagogical appropriateness of AI (50.1%) and resource and infrastructure constraints (42.3%); the burden of integrating new technology and school-level restrictions on AI use were reported by 33.3% and 13.4%, respectively. Overall, the barriers reported by non-users were concentrated in three areas: preparedness in knowledge and skills, judgments about pedagogical appropriateness, and resource conditions. Profile-specific proportions and 95% CIs for the five barriers are reported in Supplementary Table S8.

Significant between-profile differences were observed for all five barriers (all *p* < 0.001). As Figure 6 shows, reservations about the pedagogical appropriateness of AI were most pronounced in the Indifferent Profile (C1) and the Skeptical Profile (C2), at 83.3% and 67.3%, respectively, well above the Measured Endorsement Profile (C3, 36.6%) and the Risk-Aware Strong Endorsement Profile (C4, 21.8%). C3 was characterized mainly by insufficient knowledge and skills (66.2%), whereas C4 showed relatively high proportions for both insufficient knowledge and skills (58.9%) and resource and infrastructure constraints (57.1%). School-level restrictions on AI use were uncommon in all four profiles, ranging from 12.0% to 17.2%. Overall, barriers in C1 and C2 centered more on reservations about the pedagogical appropriateness of AI, whereas those in C3 and C4 centered more on knowledge and skills or resource conditions.

## 6. Discussion

### 6.1. Main Findings

This study first identified four profiles of teachers’ AI beliefs, showing that a single positive-to-negative attitude measure cannot fully capture how teachers evaluate AI. In particular, the Risk-Aware Strong Endorsement Profile combined the highest perceived AI utility with comparatively high perceived AI risk, indicating that recognition of AI’s instructional value can coexist with risk awareness. Prior research likewise shows that teachers can recognize AI’s potential value for lesson planning, delivery, and learning support while retaining concerns about reliability, ethics, and pedagogical appropriateness (Celik et al., 2022; Chounta et al., 2022; Mah & Groß, 2024). Extending this work, the present large-scale person-centered analysis across 49 education systems showed that this multidimensional evaluation is expressed not only in the relationship between the two belief dimensions but also in distinct belief configurations among teachers. The ordering of the four profiles on perceived AI utility was consistent across all 49 systems, whereas their relative ordering on perceived AI risk varied more across systems. This pattern accords with cross-national evidence of geographical and cultural variation in teachers’ judgments of AI benefits, concerns, and trust (Viberg et al., 2025). Together, the findings suggest that perceived AI utility was the primary dimension distinguishing the profiles, whereas perceived AI risk, although it provided further differentiation, may be more strongly context-dependent.

The asymmetry in the differentiating strength of the two belief dimensions further specifies the scope within which the four profiles should be interpreted. Perceived AI utility constituted the principal axis of differentiation, and perceived AI risk is better understood as a further qualification or subdivision of that axis. The four profiles should therefore be understood as empirical belief configurations within a utility-dominant structure, rather than as sharply bounded teacher types jointly defined by utility and risk to a comparable degree. Although the utility gradient is pronounced, the risk dimension still carries information that the ordering on utility alone does not capture. C2 and C3 exchange positions between the two dimensions, the ordering on perceived AI risk varies more across education systems, and perceived AI risk interacts with perceived AI utility in predicting AI use. Accordingly, the Skeptical Profile summarizes C2’s lower perceived AI utility together with perceived AI risk slightly above the overall weighted mean. The Risk-Aware Strong Endorsement Profile summarizes C4’s highest perceived AI utility together with the highest perceived AI risk among the four profiles. These labels refer to the relative positions of the profiles within the present sample and do not imply large between-profile differences on the risk dimension.

The difference between the two dimensions in cross-system stability may lie in the references against which the two judgments are made. Judgments of utility concern what AI can do in particular teaching tasks; the reference is concrete and fairly similar from one system to another. Judgments of risk are made against local institutional arrangements and normative expectations, and are therefore more likely to vary across systems. At least three kinds of system-level condition are relevant: national or school rules governing the use of AI, together with the data-protection and regulatory environment, define which practices are permitted and which consequences count as risks (Chan, 2023; Williamson & Eynon, 2020); the availability of AI tools and digital infrastructure and the extent to which AI-related professional learning is offered determine which applications teachers actually encounter and shape what their risk judgments are about (Drossel & Eickelmann, 2017; Miao & Cukurova, 2024); and the general level of public trust in AI, together with assessment culture and the degree of teacher autonomy in instructional decisions, affects how far teachers regard the involvement of AI in teaching as admissible (Bao et al., 2022; Selwyn, 2022). None of these conditions was measured here, so these explanations remain conjectures to be tested; it should also be noted that balancing the contribution of education systems is an estimation decision and does not imply that the systems themselves are interchangeable.

Second, AI professional learning showed a more consistent association with AI belief profile membership than teacher background characteristics. Prior research shows that professional learning focused on AI educational technology deepens teachers’ understanding of how such technology works and of how it can be used in teaching, and that it shifts both their trust in it and their willingness to use it (Nazaretsky et al., 2022). Cross-national research, by contrast, has found no stable association between demographic characteristics such as age, gender, or educational attainment and teachers’ trust in AI (Viberg et al., 2025). Notably, the association was strongest for the Risk-Aware Strong Endorsement Profile, suggesting that greater exposure to AI-related professional learning does not necessarily correspond to unreserved endorsement; it may accompany both higher perceived AI utility and closer scrutiny of risk. This pattern is consistent with accounts of teacher AI competence that combine pedagogical application with ethical and risk-related judgment (Celik, 2023; Tan et al., 2025). Thus, AI professional learning may relate more directly than distal background attributes to teachers’ professional judgments of AI’s instructional value and potential risks.

Third, differences between AI belief profiles extended to both AI use and pedagogical applications. AI use rates followed the same ordering as perceived AI utility, consistent with the close relationship between perceived usefulness and technology adoption that acceptance research has long emphasized (Davis, 1989; Venkatesh et al., 2003). Yet the person-centered results revealed a belief configuration obscured by average associations: the Risk-Aware Strong Endorsement Profile combined comparatively high perceived AI risk with the highest AI use rate and the highest proportions across all eight pedagogical applications. Higher perceived AI risk therefore did not necessarily correspond to less use; when perceived AI utility was high, attention to risk coexisted with higher reported use across teaching tasks. This pattern complements, rather than contradicts, the negative overall association between perceived AI risk and AI use in the variable-centered analysis: the profile analysis captures how perceived AI utility and perceived AI risk combine within the population, whereas the variable-centered model estimates their average conditional associations. Moreover, profile differences persisted among AI users, indicating that a binary measure of AI use misses meaningful variation in how AI is applied. The study therefore extends technology acceptance research beyond whether AI is adopted to the instructional tasks for which it is used, revealing a more fine-grained correspondence between heterogeneity in teachers’ AI beliefs and pedagogical practice.

It is important to distinguish the reported breadth of AI use from the quality, pedagogical appropriateness, or responsibility of that use. Preparatory uses related to content development and instructional design support planning, whereas assessment- and learning-data-related uses bear more direct implications for judgments about students and therefore require closer attention to accuracy, fairness, data protection, and teacher oversight (Hamilton et al., 2016; Selwyn, 2022; Williamson & Eynon, 2020). Across profiles, reported use was more concentrated in preparatory tasks and remained less common in assessment- and data-related tasks, but the present data cannot determine whether this pattern reflects teachers’ caution, differences in tool functionality, or access conditions. Accordingly, the Risk-Aware Strong Endorsement Profile’s higher proportions across all eight tasks indicate broader reported use, not higher-quality or more responsible AI integration.

Finally, AI non-users were also markedly heterogeneous. Insufficient knowledge and skills were the most common barrier overall, but barrier patterns differed by profile. The Indifferent and Skeptical Profiles more often reported reservations about the pedagogical appropriateness of AI. The Measured Endorsement Profile was constrained mainly by insufficient knowledge and skills, whereas the Risk-Aware Strong Endorsement Profile faced marked constraints of both knowledge and resources. This extends research identifying knowledge and skills, resource conditions, and pedagogical fit as barriers to AI use (Miao & Cukurova, 2024; Tan et al., 2025) by locating these constraints within distinct AI belief profiles. The same state of non-use can therefore reflect different conditions: some teachers remain unconvinced of AI’s pedagogical appropriateness, whereas others already endorse its utility but face insufficient knowledge and skills or resource constraints. Treating non-users as uniformly reluctant to accept AI would therefore obscure the different belief foundations and implementation conditions underlying non-use.

### 6.2. Theoretical, Methodological, and Practical Implications

Theoretically, the findings support conceptualizing teachers’ AI beliefs as a multidimensional structure comprising related but functionally distinct dimensions, rather than summarizing teachers’ judgments about AI as a single overall attitude. In particular, teachers’ evaluation of the instructional value of AI needs to be distinguished from their judgment of its potential risks, with attention to how the two are configured within the teacher population. For research across education systems, this further implies that theoretical models need to accommodate both a relatively stable common structure and context-specific components that may be shaped by institutional, cultural, and technological conditions. Research on teachers’ AI beliefs may therefore move beyond comparisons of overall attitude levels toward examining multidimensional belief configurations and their associations with instructional behavior.

Methodologically, the study indicates that person-centered analyses conducted across education systems need to attend closely to measurement comparability and to the influence of system composition on profile identification. Differences represented by latent profiles can be interpreted more confidently as substantive heterogeneity only when they rest on sufficiently comparable measurement. For large-scale international surveys, a more defensible analytic sequence is to assess cross-system measurement comparability first, then identify latent profiles, and finally examine their stability through alternative specifications, system-specific results, and sensitivity analyses. Balancing the contribution of education systems in pooled analyses can further reduce the influence of differences in system sample size on the overall pattern. This analytic logic can provide a more rigorous evidential basis for latent profile research using large cross-national databases.

In practical terms, the principal use of the AI belief profiles is to support a more differentiated identification of teachers’ needs. Barriers to AI use may involve judgments about instructional value and pedagogical appropriateness, preparedness in knowledge and skills, or resource conditions, so that teacher professional learning and school support should be differentiated in both content and pathway. Professional development can accordingly address the instructional value of AI and the boundaries of its applicability, practical competence in using AI, and judgments about its risks and ethical implications, with emphasis adjusted to the principal constraints faced by different groups of teachers.

## 7. Conclusions

Drawing on teacher data from 49 education systems in TALIS 2024, this study examined teachers’ AI beliefs from a person-centered perspective and their associations with AI-related professional learning, AI use, pedagogical applications, and barriers to AI use among non-users. Overall, teachers’ AI beliefs showed substantial heterogeneity: perceived AI utility and perceived AI risk formed distinct belief profiles, and these profiles differed in AI use, in pedagogical applications among users, and in the barriers reported by non-users. AI-related professional learning was also more consistently associated with profile membership than teacher background characteristics. Taken together, neither a single overall attitude measure nor AI use alone adequately captures the heterogeneity of teachers’ AI beliefs and practice. A fuller account requires perceived AI utility, perceived AI risk, AI-related professional learning, and the practical conditions of use and non-use to be considered together.

The limitations of the study also indicate directions for further research. First, TALIS 2024 is cross-sectional, so the associations identified here among teachers’ AI beliefs, AI-related professional learning, and AI use are concurrent; their temporal ordering and causal direction cannot be established. Longitudinal, panel, or quasi-experimental designs could examine how teachers’ AI beliefs, AI-related professional learning, and AI use change over time and how they are related to one another. Second, AI use, pedagogical applications, and barriers to AI use were all based on teacher self-reports. Although such measures are well suited to large-scale cross-system comparisons, they cannot assess the frequency, depth, or instructional quality of AI use, whether particular uses were pedagogically appropriate or responsible, or how AI use relates to actual teaching and student learning outcomes. AI use in this study should therefore be interpreted as teachers’ reported use status and range of pedagogical applications, rather than as direct evidence of high-quality integration of AI into teaching. Future research could combine classroom observations, teacher logs, interviews, platform behavioral data, and student learning outcomes to provide more direct evidence of how AI is used in teaching. AI-related professional learning was likewise measured as a binary indicator of participation during the previous 12 months, a coding that records neither the intensity nor the content of that participation. Because this variable was the correlate most consistently associated with profile membership, the present findings indicate that exposure to such professional learning matters, but not what kind of provision matters. Measures that capture duration, format, and content would be needed to address that question. Third, the TALIS 2024 items measure the content of teachers’ beliefs about the utility and risks of AI rather than any conflict or hesitation that teachers subjectively experience. The profiles identified here should therefore be read as configurations of belief content. Whether a simultaneously high evaluation of utility and of risk is accompanied by subjectively experienced conflict, and whether it corresponds to more deliberate weighing and greater context dependence, requires measures that the present data do not contain. Fourth, TALIS 2024 adopts a broad definition of AI, and the relevant items do not specify a particular tool or application context; teachers may therefore have referred to different forms of AI when responding. Because these applications may differ in instructional function, data use, and potential risk, the identified belief structure and profiles reflect teachers’ overall appraisals of the broad category of AI covered by TALIS, and their applicability to specific tools and contexts requires further examination. Fifth, the study included the 49 education systems in TALIS 2024 that administered the relevant AI items and met the analytic requirements; the findings should therefore not be generalized directly to all countries or regions. Although cross-system measurement comparability checks and sensitivity analyses supported the stability of the main findings, education systems still differed in perceived AI risk and profile distributions. The profiles identified here should therefore be understood as empirical patterns observed across these 49 education systems, rather than as teacher types with identical characteristics or proportions in every system. Future research could replicate the analysis in a wider range of educational contexts and directly measure the regulatory, provisioning, and cultural conditions discussed above, in order to explain cross-system differences in the profiles and in their behavioral associations.

## Figures and Tables

**Figure 1:**
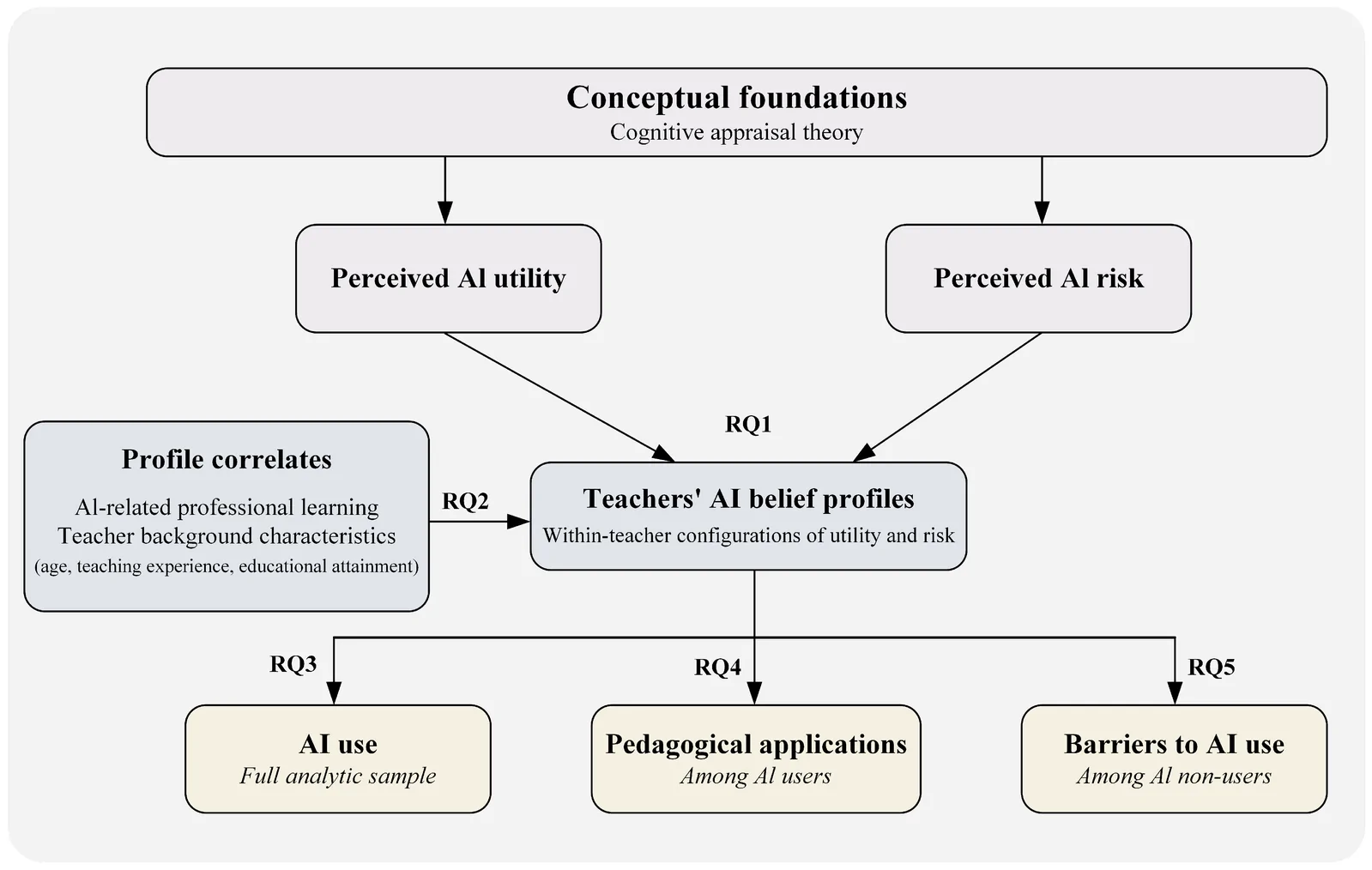
Conceptual and Analytic Framework of the Study. Note. Arrows indicate the conceptual and analytic relationships corresponding to RQ1–RQ5.

**Figure 2:**
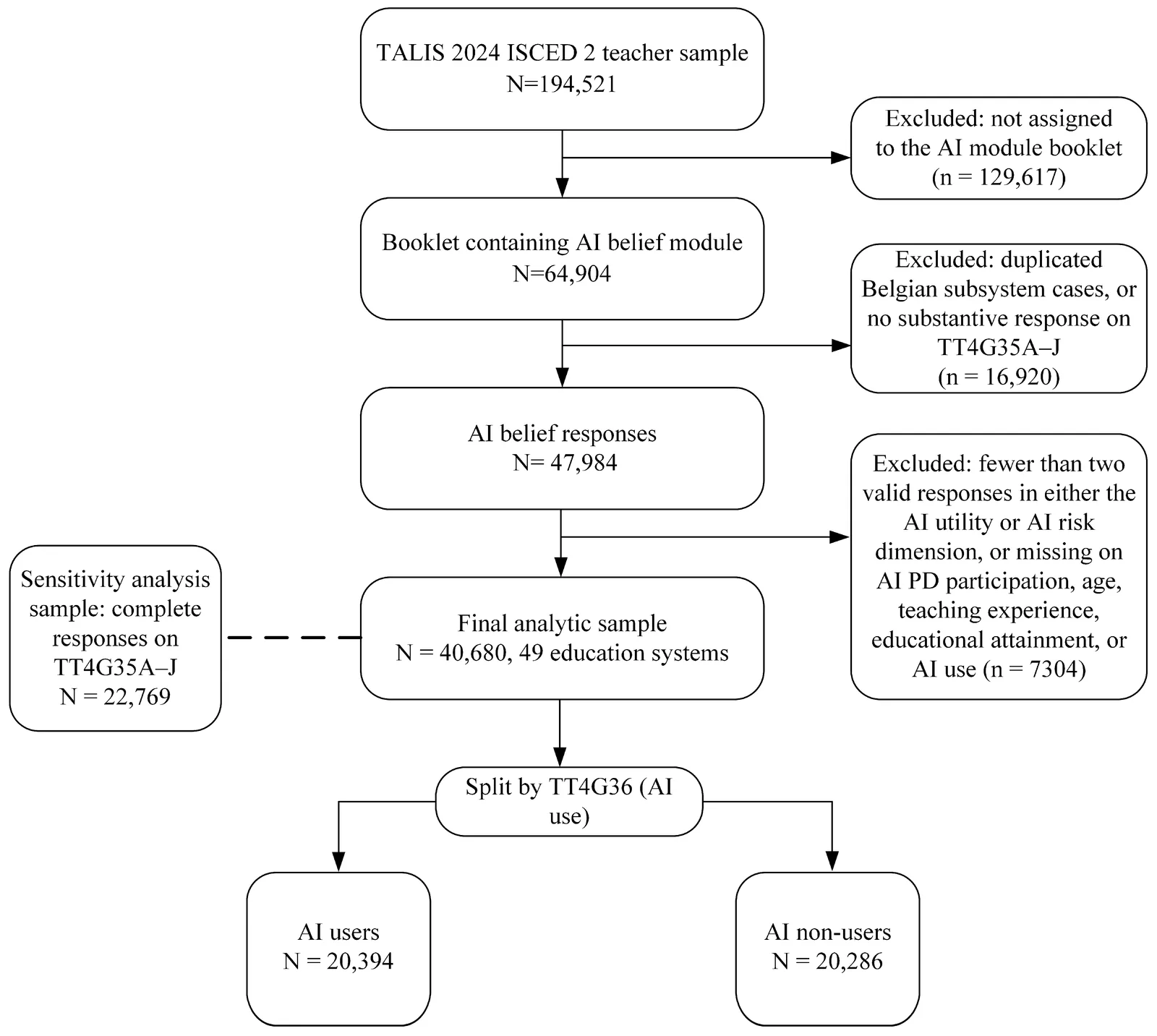
Sample Selection Flowchart. Note. Solid arrows indicate the main sample-selection flow, whereas the dashed line indicates the complete-response subsample used for sensitivity analysis.

**Figure 3:**
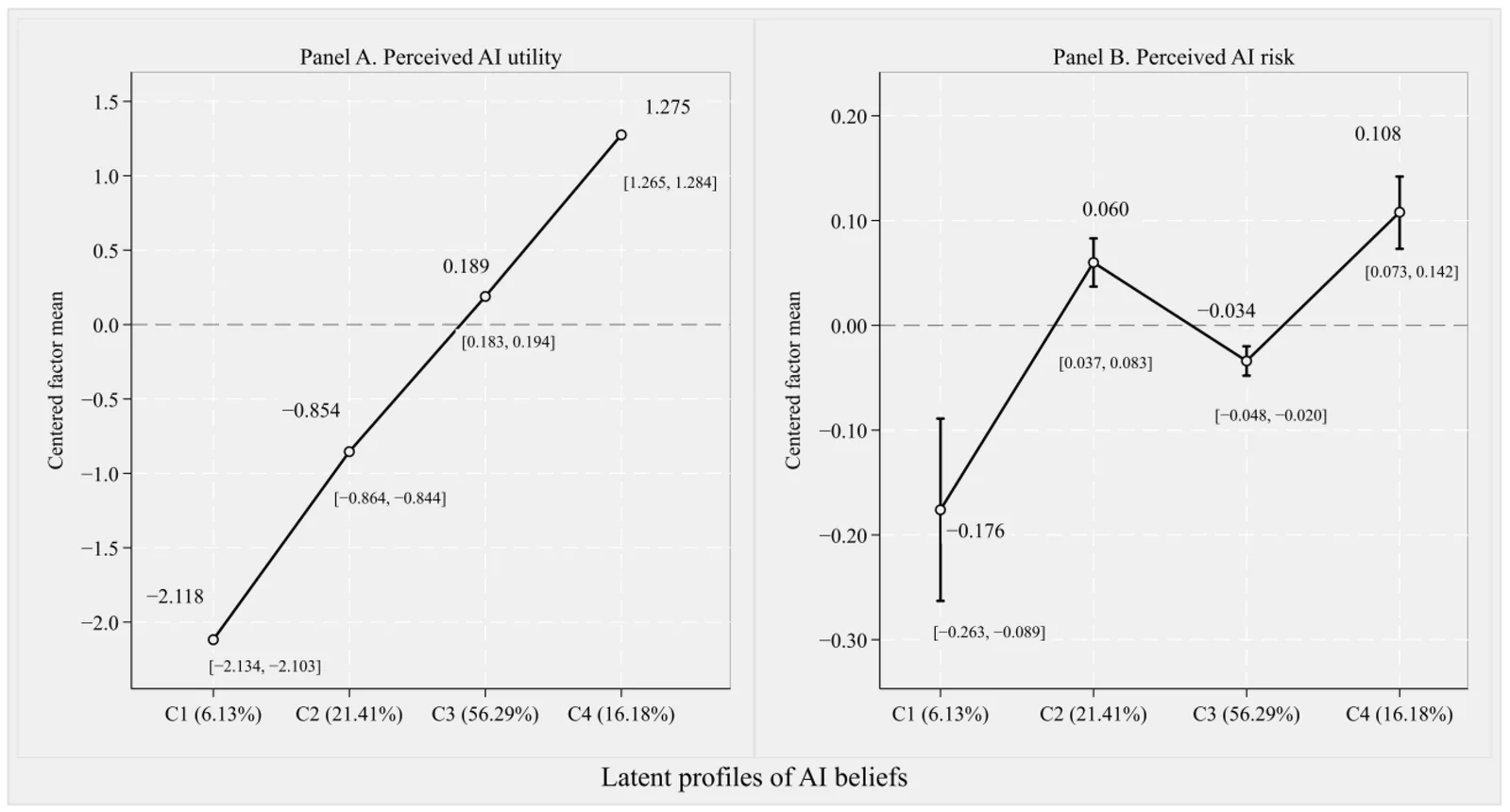
Latent Profiles of Teachers’ AI Beliefs. Note. The zero line indicates the overall weighted mean across the 49 education systems, error bars indicate 95% CIs, and percentages in parentheses indicate profile proportions. C1 = Indifferent Profile; C2 = Skeptical Profile; C3 = Measured Endorsement Profile; C4 = Risk-Aware Strong Endorsement Profile.

**Figure 4:**
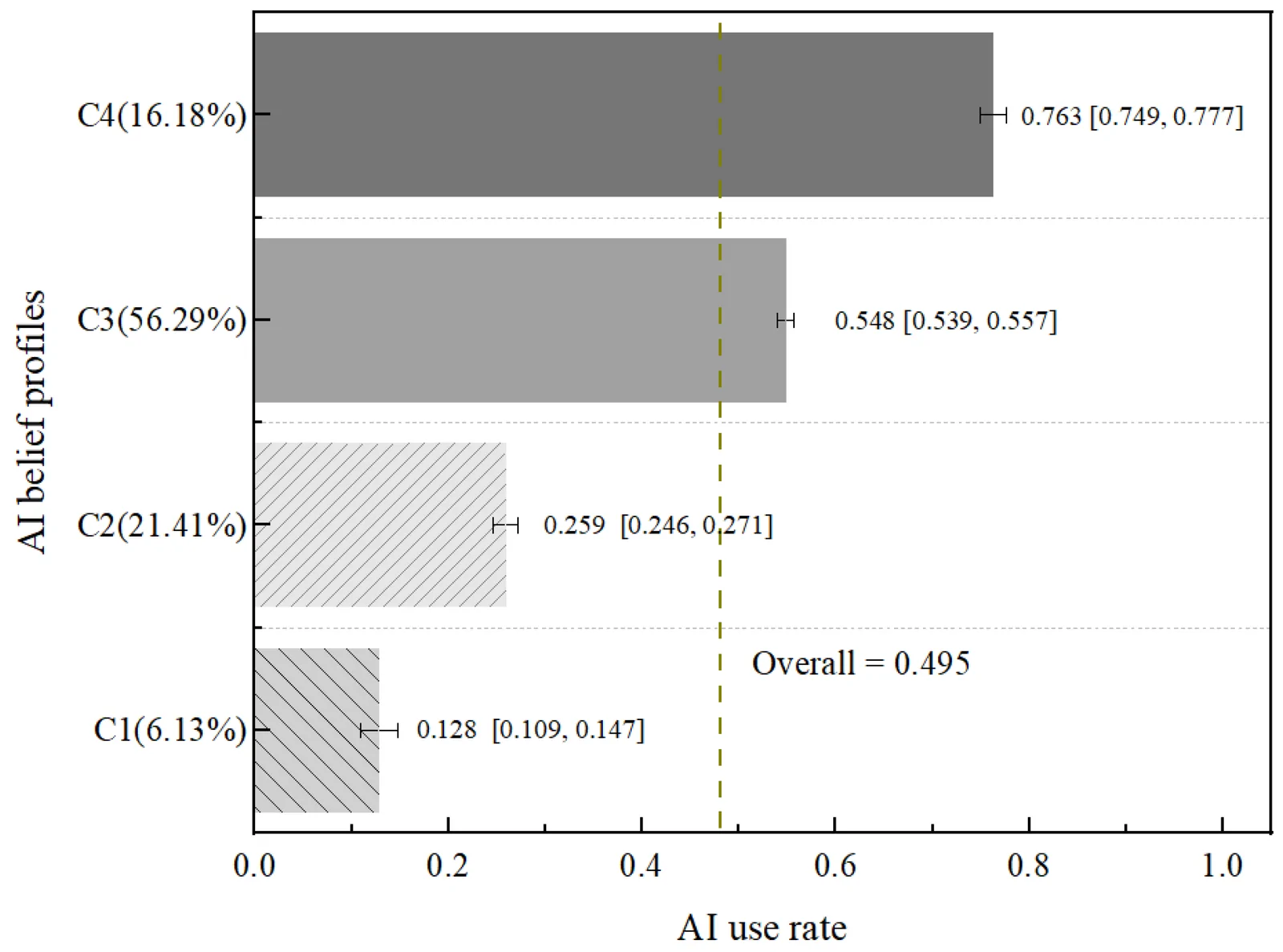
AI Use Rates by AI Belief Profiles. Note. Error bars indicate 95% CIs; the dashed line indicates the overall weighted AI use rate (49.5%); percentages in parentheses indicate profile proportions. 95% CIs were estimated using Taylor series linearization with adjustment for school-level clustering. C1 = Indifferent Profile; C2 = Skeptical Profile; C3 = Measured Endorsement Profile; C4 = Risk-Aware Strong Endorsement Profile.

**Figure 5:**
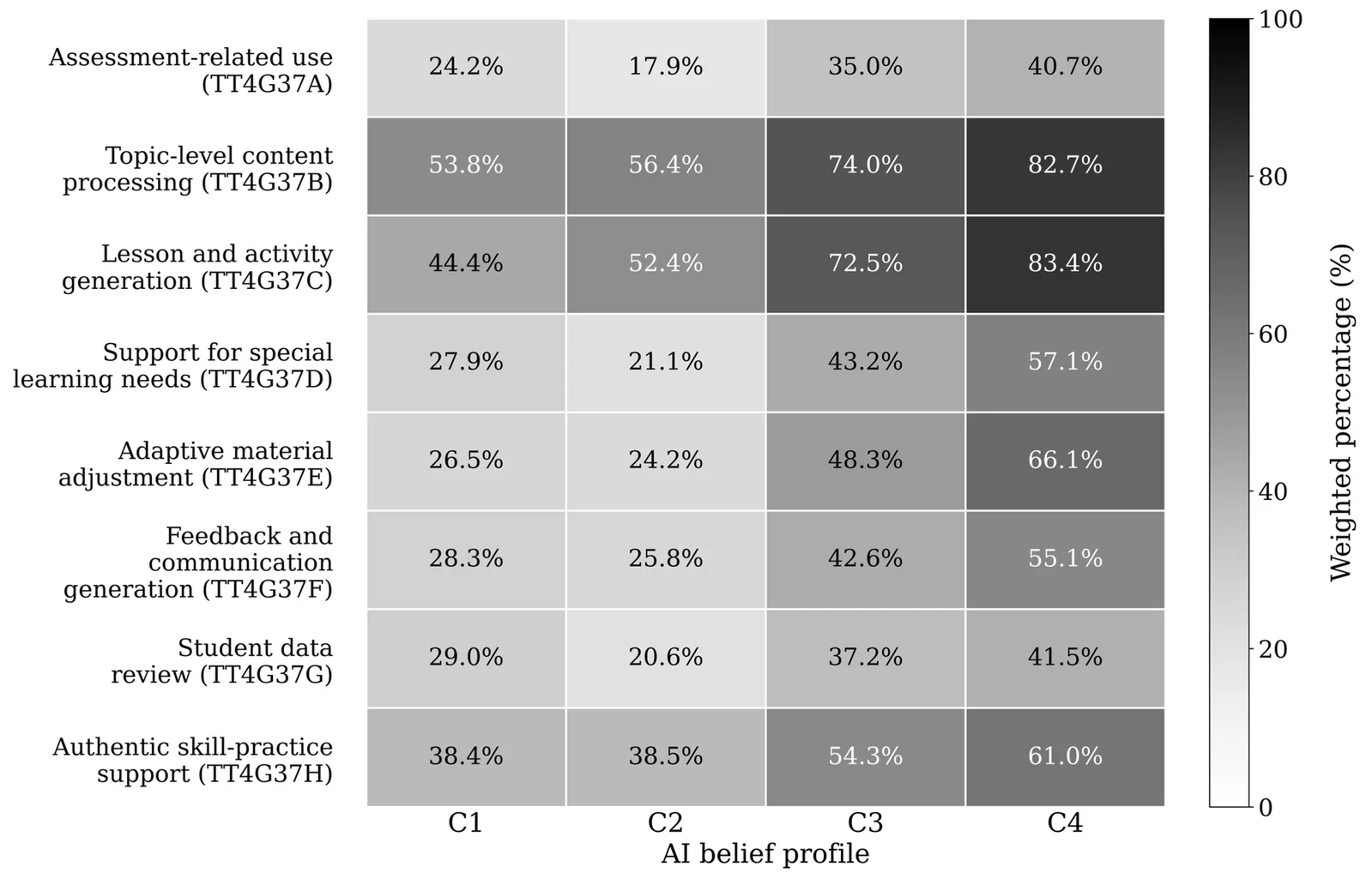
Eight Pedagogical Applications of AI by AI Belief Profile Among AI Users (N = 20,394). Note. Cells show the weighted percentage of teachers within each profile who reported the corresponding pedagogical application; darker shading indicates a higher percentage. The corresponding 95% CIs are reported in Supplementary Table S7. C1 = Indifferent Profile; C2 = Skeptical Profile; C3 = Measured Endorsement Profile; C4 = Risk-Aware Strong Endorsement Profile.

**Figure 6:**
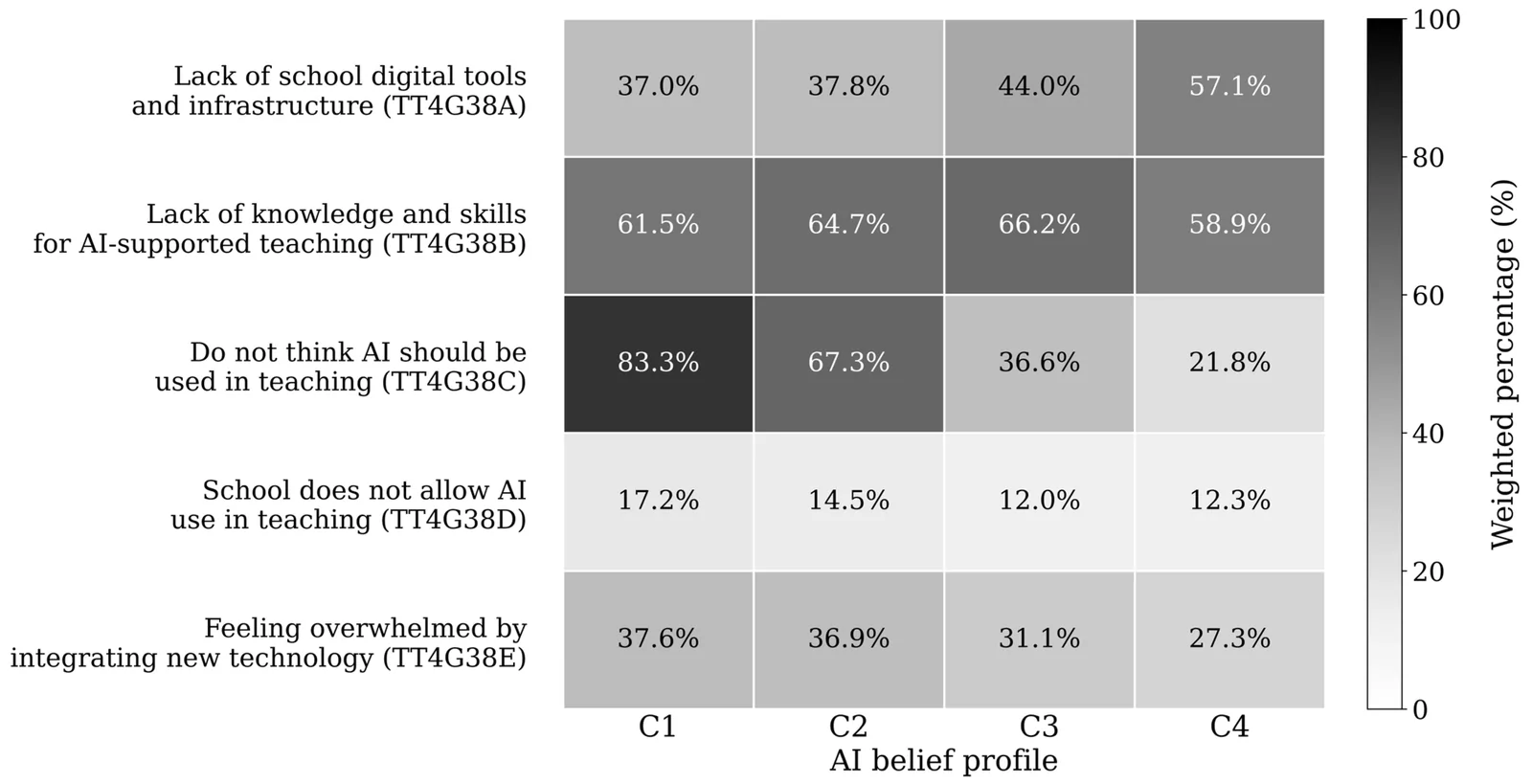
Five Barriers to AI Use by AI Belief Profile Among Non-Users (N = 20,286). Note. Cells show the weighted percentage of teachers within each profile who reported the corresponding barrier to AI use; darker shading indicates a higher percentage. The corresponding 95% CIs are reported in Supplementary Table S8. C1 = Indifferent Profile; C2 = Skeptical Profile; C3 = Measured Endorsement Profile; C4 = Risk-Aware Strong Endorsement Profile.

**Table 1:** Descriptive Statistics for the Teacher Sample (N = 40,680).

Variable	Category	*n*	%
AI professional learning	No participation	21,002	51.63
	Participation	19,678	48.37
Age	≤29	4954	12.18
	30–39	10,871	26.72
	40–49	12,731	31.30
	50–59	8950	22.00
	≥60	3174	7.80
Teaching experience	≤5	8439	20.74
	6–10	6945	17.07
	11–20	11,904	29.26
	>20	13,392	32.92
Highest educational attainment	Upper secondary education or below	685	1.68
	Short-cycle tertiary education	1125	2.77
	Bachelor	20,478	50.34
	Master	17,723	43.57
	Doctoral	669	1.64

**Table 2:** Fit Indices for Latent Profile Models of Teachers’ AI Belief Factor Scores Across 49 Education Systems (N = 40,680).

k	LL	AIC	BIC	SSA-BIC	ΔBIC	Entropy	LMR p	Profile Proportions
2	−101,851.314	203,716.628	203,776.922	203,754.676	—	0.985	<0.001	0.017/0.983
3	−100,340.653	200,701.307	200,787.442	200,755.662	2989.480	0.892	<0.001	0.018/0.900/0.082
**4**	**−94** **,** **917.371**	**189** **,** **860.743**	**189** **,** **972.718**	**189** **,** **931.404**	**10** **,** **814.724**	**0** **.941**	**<** **0** **.001**	**0** **.162/** **0** **.214/** **0** **.061/** **0** **.563**
5	−93,005.150	186,042.301	186,180.117	186,129.269	3792.601	0.947	<0.001	0.162/0.016/0.046/0.214/0.562
6	−92,325.496	184,688.992	184,852.648	184,792.266	1327.469	0.918	<0.001	0.046/0.016/0.561/0.079/0.085/0.214

Note. *k* = number of profiles; LL = log-likelihood; AIC = Akaike information criterion; BIC = Bayesian information criterion; SSA-BIC = sample-size adjusted BIC; ΔBIC = reduction in BIC relative to the preceding model; LMR = Lo–Mendell–Rubin likelihood ratio test. Bold indicates the retained model.

**Table 3:** Descriptive and Inferential Statistics for the Four AI Belief Profiles (N = 40,680).

**Panel A. Profile-Specific Descriptive Statistics**						
**AI Belief Dimension**	**C1 (6.13%)** ***M* [95% CI]**	**C2 (21.41%)** ***M* [95% CI]**		**C3 (56.29%)** ***M* [95% CI]**	**C4 (16.18%)** ***M* [95% CI]**	***SD***
Perceived AI utility	−2.118 [−2.134, −2.103]	−0.854 [−0.864, −0.844]		0.189 [0.183, 0.194]	1.275 [1.265, 1.284]	0.253
Perceived AI risk	−0.176 [−0.263, −0.089]	0.060 [0.037, 0.083]		−0.034 [−0.048, −0.020]	0.108 [0.073, 0.142]	0.846
**Panel B. Between-Profile Comparisons**						
**AI Belief Dimension**	**Wald χ^2^(3)**	***p***	**Holm-Adjusted Profile Ordering**		**Pairwise Cohen’s |*d*| Range**	
Perceived AI utility	164,209.924	<0.001	C4 > C3 > C2 > C1		4.123–13.416	
Perceived AI risk	106.178	<0.001	C4 > C2 > C3 > C1		0.056–0.335	

Note. *M* = mean; *SD* = standard deviation; CI = confidence interval. Panel A reports profile means with 95% CIs, based on factor scores centered so that 0 represents the overall weighted mean across the 49 education systems. Within-profile variances were constrained to be equal across profiles within each belief dimension, so a single SD is reported for each dimension. Panel B reports the overall Wald test, the profile ordering obtained from Holm-adjusted pairwise comparisons, and the range of absolute Cohen’s |d| values across the six pairwise comparisons. C1 = Indifferent Profile; C2 = Skeptical Profile; C3 = Measured Endorsement Profile; C4 = Risk-Aware Strong Endorsement Profile.

**Table 4:** R3STEP Multinomial Logistic Regression Results for AI Belief Profile Membership (N = 40,680).

Variable	C2 vs. C1			C3 vs. C1			C4 vs. C1		
	*OR*	*SE*	95% CI	*OR*	*SE*	95% CI	*OR*	*SE*	95% CI
AI professional learning									
Participation	1.607 ***	0.085	[1.360, 1.898]	3.113 ***	0.079	[2.668, 3.636]	5.856 ***	0.090	[4.907, 6.982]
Age									
30–39	0.973	0.160	[0.711, 1.332]	1.043	0.149	[0.779, 1.397]	0.928	0.167	[0.669, 1.287]
40–49	0.856	0.169	[0.615, 1.193]	0.927	0.160	[0.677, 1.268]	0.772	0.182	[0.540, 1.103]
50–59	1.046	0.185	[0.728, 1.503]	1.083	0.174	[0.770, 1.524]	0.818	0.206	[0.546, 1.225]
60 or older	1.076	0.206	[0.719, 1.612]	1.265	0.197	[0.860, 1.861]	1.016	0.235	[0.641, 1.611]
Teaching experience									
6–10 years	0.790	0.132	[0.610, 1.023]	0.767 *	0.124	[0.602, 0.978]	0.755 *	0.142	[0.572, 0.997]
11–20 years	0.808	0.127	[0.629, 1.036]	0.770 *	0.117	[0.612, 0.968]	0.615 ***	0.139	[0.468, 0.808]
>20 years	0.834	0.142	[0.631, 1.101]	0.843	0.133	[0.649, 1.094]	0.626 **	0.162	[0.456, 0.860]
Highest educational attainment									
Upper secondary or below	0.439 **	0.266	[0.260, 0.739]	0.694	0.239	[0.435, 1.109]	0.867	0.281	[0.500, 1.505]
Short-cycle tertiary	0.770	0.220	[0.500, 1.186]	0.792	0.207	[0.528, 1.189]	0.911	0.235	[0.575, 1.444]
Master	0.876	0.096	[0.726, 1.058]	0.810 *	0.090	[0.679, 0.967]	0.863	0.101	[0.708, 1.051]
Doctoral	0.640	0.234	[0.405, 1.013]	0.546 **	0.224	[0.352, 0.847]	0.853	0.264	[0.508, 1.431]

Note. *OR* = odds ratio; *SE* = standard error of the logit coefficient. The reference categories for age, teaching experience, educational attainment, and AI-related professional learning were 29 years or younger, five years or less, bachelor’s degree, and no participation, respectively. C1 = Indifferent Profile; C2 = Skeptical Profile; C3 = Measured Endorsement Profile; C4 = Risk-Aware Strong Endorsement Profile. *** *p* < 0.001, ** *p* < 0.01, * *p* < 0.05.

## Data Availability

Publicly available data were analyzed in this study. The TALIS 2024 public use files are available from the OECD TALIS 2024 Database at https://www.oecd.org/en/data/datasets/talis-2024-database.html (accessed on 12 May 2026).

## Supplementary Materials

The following supporting information can be downloaded at: https://www.mdpi.com/article/10.3390/bs16101762/s1, Figure S1: Forest plots of the associations of perceived AI utility and perceived AI risk with AI use across 49 education systems; Table S1: Fixed alignment diagnostics of approximate measurement invariance for the AI belief items, and reference-system sensitivity; Table S2: Four-profile sensitivity analysis among teachers with complete responses to all AI belief items (N = 22,769); Table S3: AI belief profile proportions and mean perceived AI utility (raw 1–4 metric) across 49 education systems; Table S4: Mean perceived AI risk (raw 1–4 metric) and profile-size diagnostics across 49 education systems; Table S5: Associations of perceived AI utility and perceived AI risk with teachers’ AI use; Table S6: System-specific binary logistic regressions of AI use on perceived AI utility and perceived AI risk (49 education systems); Table S7: Weighted proportions and 95% confidence intervals for eight pedagogical applications of AI among AI users; Table S8: Weighted proportions and 95% confidence intervals for five barriers to AI use among non-users.
