# Supplementary material for: Teachers’ AI Belief Profiles and Their Associations with AI Use, Pedagogical Applications, and Barriers: A Person-Centered Analysis of TALIS 2024
Source: Behav Sci (Basel). 2026 Sep 28;16(10):1762. doi: 10.3390/bs16101762 (PMC13701813; doi:10.3390/bs16101762)
Supplement: Supplementary file 1 [file behavsci-16-01762-s001.zip › behavsci-4550739-supplementary.pdf]

Table S1 Fixed alignment diagnostics of approximate measurement invariance for the AI belief items, and reference-system sensitivity

Panel A. Measurement invariance diagnostics

Dimension	Item	724 reference system		398 reference system	
		Intercept	Loading	Intercept	Loading
Perceived AI utility	TT4G35A	15	1	17	1
	TT4G35B	6	0	12	0
	TT4G35C	8	1	7	1
	TT4G35D	14	0	15	0
	TT4G35E	10	1	16	1
Perceived AI risk	TT4G35F	10	4	14	4
	TT4G35G	12	6	16	6
	TT4G35H	6	0	3	0
	TT4G35I	10	0	18	0
	TT4G35J	14	4	14	4
Perceived AI utility subtotal	—	53/245 (21.6%)	3/245 (1.2%)	67/245 (27.3%)	3/245 (1.2%)
Perceived AI risk subtotal	—	52/245 (21.2%)	14/245 (5.7%)	65/245 (26.5%)	14/245 (5.7%)
Overall	—	105/490 (21.4%)	17/490 (3.5%)	132/490 (26.9%)	17/490 (3.5%)

Panel B. Reference-system sensitivity

Robustness indicator	Result
Hard-classification agreement	99.62%
Adjusted Rand index (ARI)	0.989
C1 posterior-probability correlation	0.9998
C2 posterior-probability correlation	0.9996
C3 posterior-probability correlation	0.9998
C4 posterior-probability correlation	0.9996
Perceived AI utility factor-score correlation	0.9999
Perceived AI risk factor-score correlation	0.9977

Note. C1 = Indifferent Profile; C2 = Skeptical Profile; C3 = Measured Endorsement Profile; C4 = Risk-Aware Strong Endorsement Profile. Item rows report the number of education systems in which the corresponding parameter was identified as non-invariant by fixed alignment. The Subtotal and Overall rows report both the number of non-invariant parameters and their percentage of the corresponding parameter total. The five items for perceived AI utility and the five items for perceived AI risk each correspond to 245 intercept parameters and 245 loading parameters across the 49 education systems. Spain (724) was used as the reference system in the main analysis, and Kazakhstan (398) as the alternative reference system in the sensitivity analysis. Across all intercept and loading parameters, 122/980 (12.4%) were identified as non-invariant with 724 as the reference system, compared with 149/980 (15.2%) with 398 as the reference system. Fixed alignment was estimated using MLR, with the four-point items treated as continuous indicators.

Table S2 Four-profile sensitivity analysis among teachers with complete responses to all AI belief items (N = 22,769)

Sensitivity-model class	Corresponding profile	Proportion (%)	Mean utility factor score	Mean risk factor score
C1	Indifferent Profile	5.90	−1.845	−1.040
C3	Skeptical Profile	20.75	−0.528	−0.521
C4	Measured Endorsement Profile	55.69	0.531	−0.516
C2	Risk-Aware Strong Endorsement Profile	17.65	1.636	−0.322

Note. LL = −54,294.543, AIC = 108,615.085, BIC = 108,719.516, SSA-BIC = 108,678.203, and entropy = 0.950. Class numbering in the sensitivity model differs from that in the main model; classes are matched here to the main-model profiles according to their mean perceived AI utility and perceived AI risk factor scores. The complete-response analysis recovered four substantively comparable profiles with proportions similar to those in the main model.

Table S3 AI belief profile proportions and mean perceived AI utility (raw 1–4 metric) across 49 education systems

System ID	N	C1 (%)	C2 (%)	C3 (%)	C4 (%)	C1 mean	C2 mean	C3 mean	C4 mean
31	1155	1.21	10.19	73.97	14.64	1.068	2.121	3.013	3.751
36	653	3.66	19.42	58.27	18.65	1.121	2.196	2.956	3.809
48	815	2.71	8.53	64.16	24.61	1.139	2.196	3.006	3.805
56	964	10.72	28.61	50.13	10.54	1.164	2.141	2.902	3.707
76	783	3.83	17.04	64.87	14.27	1.183	2.163	2.931	3.797
100	754	9.88	31.01	50.78	8.33	1.166	2.135	2.936	3.779
152	609	3.13	13.39	55.21	28.26	1.227	2.163	2.953	3.786
170	811	1.81	16.31	58.67	23.21	1.215	2.173	2.946	3.824
188	729	5.24	13.35	52.35	29.05	1.236	2.198	2.966	3.825
191	774	7.38	33.15	51.46	8.01	1.274	2.166	2.932	3.728
196	424	5.21	14.96	62.89	16.95	1.092	2.194	2.981	3.784
203	1472	2.67	19.77	65.51	12.05	1.187	2.189	2.940	3.691
208	441	15.55	41.84	36.57	6.04	1.252	2.181	2.937	3.750
233	774	3.52	23.85	63.23	9.40	1.109	2.180	2.934	3.786
246	750	7.04	35.85	52.40	4.71	1.193	2.206	2.911	3.783
250	494	34.26	37.88	23.75	4.11	1.177	2.166	2.977	3.840
348	729	9.97	29.17	51.39	9.47	1.194	2.139	2.935	3.735
352	232	3.92	22.91	54.65	18.52	1.226	2.108	2.977	3.858
376	625	6.45	23.34	52.27	17.94	1.144	2.208	2.948	3.830
380	930	8.80	34.64	52.18	4.39	1.237	2.198	2.937	3.739
398	2039	1.37	10.21	71.74	16.68	1.139	2.169	2.991	3.761
410	904	1.64	12.61	65.42	20.33	1.073	2.251	2.978	3.770
411	691	3.67	22.35	57.81	16.17	1.235	2.156	3.013	3.754
428	900	3.39	30.22	59.93	6.46	1.218	2.181	2.939	3.691
440	910	3.02	24.93	60.90	11.15	1.240	2.187	2.952	3.786
470	489	1.84	13.95	60.90	23.31	1.062	2.198	2.961	3.793
499	296	13.85	30.17	46.98	9.00	1.228	2.164	2.930	3.834
504	1326	5.74	21.31	57.09	15.85	1.228	2.158	2.974	3.790
528	240	13.39	28.96	41.78	15.87	1.223	2.255	2.889	3.747
554	269	2.79	10.88	58.36	27.98	1.116	2.194	2.922	3.723
578	289	9.24	28.61	50.09	12.06	1.291	2.215	2.940	3.796
616	888	3.30	20.59	67.60	8.51	1.212	2.164	2.980	3.776
620	790	8.10	30.26	54.48	7.16	1.254	2.196	2.940	3.734
682	722	1.27	5.07	64.48	29.18	1.004	2.190	3.006	3.838
688	964	12.81	31.00	48.08	8.11	1.158	2.178	2.962	3.797
703	921	4.43	26.63	62.12	6.83	1.163	2.170	2.942	3.735
704	1388	0.62	6.43	66.46	26.48	1.214	2.235	2.983	3.797
705	696	7.89	38.51	48.45	5.15	1.204	2.184	2.911	3.775
710	790	1.77	6.50	56.03	35.70	1.091	2.109	3.014	3.793
724	1993	11.49	30.25	47.12	11.13	1.158	2.131	2.904	3.788
752	446	13.12	28.29	50.97	7.62	1.136	2.141	2.900	3.768
784	1259	1.33	6.22	59.61	32.85	1.278	2.253	2.986	3.837
792	1299	2.77	6.54	57.86	32.84	1.166	2.169	3.001	3.848
807	975	6.95	26.41	54.06	12.58	1.159	2.133	2.962	3.802
840	372	6.26	17.85	51.38	24.52	1.096	2.157	2.985	3.814

System ID	N	C1 (%)	C2 (%)	C3 (%)	C4 (%)	C1 mean	C2 mean	C3 mean	C4 mean
860	1313	2.29	10.80	63.23	23.69	1.136	2.123	3.026	3.775
9134	395	3.36	10.94	62.08	23.62	1.144	2.239	3.001	3.816
9642	941	4.18	27.54	53.82	14.47	1.139	2.168	2.951	3.784
156001	1257	1.71	9.57	64.61	24.11	1.288	2.224	2.963	3.814

Note. N denotes the unweighted analytic sample size; profile proportions and means are weighted estimates, with means reported on the raw four-point response metric (1–4). Across all 49 education systems, the four profiles showed the same ordering in perceived AI utility: C1 < C2 < C3 < C4. C1 = Indifferent Profile; C2 = Skeptical Profile; C3 = Measured Endorsement Profile; C4 = Risk-Aware Strong Endorsement Profile.

Table S4 Mean perceived AI risk (raw 1–4 metric) and profile-size diagnostics across 49 education systems

System ID	N	C1	C2	C3	C4	Risk range	Highest-risk profile	Minimum expected profile n
31	1155	1.582	2.457	2.519	2.832	1.249	C4	15.219
36	653	3.115	3.271	3.185	3.017	0.255	C2	24.237
48	815	2.126	2.838	2.847	2.965	0.840	C4	23.093
56	964	2.733	2.874	2.926	2.984	0.251	C4	95.400
76	783	2.667	2.908	2.757	2.780	0.241	C2	29.868
100	754	2.851	2.976	2.903	2.993	0.143	C4	58.275
152	609	2.041	2.980	2.830	2.900	0.938	C2	18.992
170	811	2.450	2.745	2.791	2.987	0.537	C4	16.161
188	729	2.436	2.995	2.895	3.062	0.626	C4	34.433
191	774	3.065	2.941	2.786	2.862	0.279	C1	64.497
196	424	1.805	2.916	2.822	2.813	1.111	C2	18.617
203	1472	2.863	3.025	2.940	2.745	0.280	C2	39.027
208	441	3.099	2.980	3.098	3.030	0.119	C1	29.091
233	774	2.881	3.032	3.075	3.136	0.255	C4	27.857
246	750	3.162	3.147	3.076	2.928	0.234	C1	37.367
250	494	2.965	3.154	3.068	2.758	0.396	C2	18.158
348	729	2.705	2.945	2.875	2.945	0.240	C2	64.462
352	232	3.183	2.807	2.959	2.885	0.376	C1	10.212
376	625	2.250	2.863	2.908	3.186	0.936	C4	28.753
380	930	2.869	2.883	2.724	2.447	0.435	C2	43.835
398	2039	1.799	2.645	2.545	2.649	0.850	C4	25.436
410	904	2.028	2.841	2.935	3.100	1.072	C4	12.360
411	691	2.702	2.847	2.816	2.904	0.203	C4	27.308
428	900	2.942	2.994	2.943	3.091	0.149	C4	30.147
440	910	2.582	2.977	2.937	2.977	0.395	C4	28.424
470	489	2.171	2.919	2.975	3.144	0.973	C4	10.105
499	296	2.812	2.923	2.816	3.073	0.260	C4	25.907
504	1326	2.450	2.997	3.017	3.054	0.604	C4	68.686
528	240	3.121	2.871	3.011	3.068	0.250	C1	21.664
554	269	3.347	3.326	3.222	3.026	0.321	C1	7.495
578	289	3.144	3.138	3.249	3.313	0.175	C4	26.135
616	888	3.163	2.876	2.918	2.962	0.287	C1	26.810
620	790	2.853	3.076	2.964	2.909	0.223	C2	54.926
682	722	1.622	2.682	2.791	3.026	1.404	C4	9.026
688	964	2.385	2.853	2.827	2.968	0.583	C4	78.865
703	921	2.596	2.985	3.005	3.005	0.410	C3	43.480

System ID	N	C1	C2	C3	C4	Risk range	Highest-risk profile	Minimum expected profile n
704	1388	1.595	2.387	2.468	2.697	1.102	C4	9.786
705	696	2.961	2.855	2.792	2.861	0.170	C1	33.248
710	790	1.931	2.771	2.807	2.908	0.977	C4	17.956
724	1993	2.836	3.173	3.142	3.198	0.362	C4	219.253
752	446	3.016	3.216	3.112	3.099	0.200	C2	39.771
784	1259	2.735	3.000	2.850	2.923	0.265	C2	18.305
792	1299	2.032	2.710	2.943	3.120	1.088	C4	32.974
807	975	2.624	2.880	2.877	3.029	0.404	C4	70.435
840	372	3.328	3.084	2.977	3.075	0.350	C1	22.204
860	1313	1.931	2.490	2.502	2.675	0.745	C4	34.180
9134	395	3.272	3.426	3.124	3.047	0.378	C2	12.311
9642	941	2.887	2.869	2.786	2.759	0.128	C1	39.133
156001	1257	2.169	2.702	2.758	2.962	0.793	C4	22.137

Note. N denotes the unweighted analytic sample size; profile means are weighted estimates on the raw four-point response metric (1–4). Within each education system, the range of profile means in perceived AI risk averaged 0.507 (median 0.376, maximum 1.404), and the smallest expected profile size in any system was 7.50. The profile with the highest perceived AI risk varied across education systems (C4 in 26 systems, C2 in 12, C1 in 10, and C3 in 1). The highest-risk profile was identified from the unrounded estimates; its variation across systems indicates cross-system heterogeneity in how the profiles differ on perceived AI risk. C1 = Indifferent Profile; C2 = Skeptical Profile; C3 = Measured Endorsement Profile; C4 = Risk-Aware Strong Endorsement Profile.

Table S5 Associations of perceived AI utility and perceived AI risk with teachers' AI use

Panel A. Pooled-sample binary logistic regression

Predictor	b	SE	OR	95% CI	p
Model 1. Main effects					
Perceived AI utility	1.008	0.025	2.740	[2.611, 2.874]	< 0.001
Perceived AI risk	−0.198	0.020	0.820	[0.788, 0.853]	< 0.001
Model 2. Adding perceived AI utility × perceived AI risk					
Perceived AI utility	0.987	0.026	2.684	[2.549, 2.825]	< 0.001
Perceived AI risk	−0.184	0.022	0.832	[0.797, 0.869]	< 0.001
Perceived AI utility × perceived AI risk	−0.050	0.020	0.952	[0.914, 0.990]	0.015

Panel B. System-specific estimates across 49 education systems and random-effects summary

Predictor	Direction across systems	Significant systems	Random-effects OR [95% CI]	τ^2	I^2 (%)	Q(48)	Heterog.p
Perceived AI utility	Positive 49/49; negative 0/49	49/49	2.866 [2.604, 3.155]	0.0900	80.08	248.06	< 0.001
Perceived AI risk	Negative 40/49; positive 9/49	16/49	0.844 [0.800, 0.889]	0.0170	52.07	98.07	< 0.001

Note. Panel A reports a pooled-sample binary logistic regression adjusted for AI-related professional learning, age, teaching experience, highest educational attainment, and education-system fixed effects. TCHWGT was rescaled to sum to 1 within each education system, giving every system the same total weight in the pooled model; standard errors were adjusted for clustering at the school level. Panel B reports estimates from the same model fitted separately in the 49 education systems and their restricted maximum-likelihood (REML) random-effects summary. Within Panel A, Model 1 includes the two AI belief dimensions as main effects, and Model 2 additionally includes their interaction; the two models use the same covariates, weighting, and clustering. Panel B corresponds to the Model 1 specification estimated separately within each education system.

Table S6 System-specific binary logistic regressions of AI use on perceived AI utility and perceived AI risk (49 education systems)

System ID	N	No. of schools	Utility OR [95% CI]	p	Risk OR [95% CI]	p
31	1155	221	1.949 [1.476, 2.575]	< 0.001	0.883 [0.741, 1.053]	0.166
36	653	177	4.162 [2.993, 5.787]	< 0.001	1.397 [1.024, 1.907]	0.035
48	815	116	1.589 [1.220, 2.070]	< 0.001	0.690 [0.567, 0.841]	< 0.001
56	964	269	3.303 [2.571, 4.244]	< 0.001	0.676 [0.531, 0.862]	0.002
76	783	198	3.264 [2.367, 4.503]	< 0.001	1.074 [0.831, 1.388]	0.583
100	754	197	2.876 [2.126, 3.890]	< 0.001	0.724 [0.573, 0.915]	0.007
152	609	317	2.692 [1.842, 3.933]	< 0.001	0.757 [0.559, 1.026]	0.073
170	811	203	2.580 [1.862, 3.575]	< 0.001	0.673 [0.507, 0.893]	0.006
188	729	183	2.339 [1.816, 3.012]	< 0.001	0.598 [0.473, 0.756]	< 0.001
191	774	259	3.299 [2.355, 4.623]	< 0.001	0.913 [0.689, 1.209]	0.524
196	424	98	4.201 [2.863, 6.165]	< 0.001	0.599 [0.417, 0.859]	0.006
203	1472	341	3.339 [2.640, 4.224]	< 0.001	0.725 [0.594, 0.885]	0.002
208	441	168	3.006 [2.210, 4.088]	< 0.001	0.781 [0.565, 1.078]	0.132
233	774	198	2.163 [1.582, 2.956]	< 0.001	0.949 [0.717, 1.256]	0.712
246	750	224	3.525 [2.509, 4.953]	< 0.001	1.230 [0.869, 1.742]	0.242
250	494	152	2.131 [1.609, 2.823]	< 0.001	1.101 [0.831, 1.457]	0.501
348	729	220	3.848 [2.798, 5.293]	< 0.001	1.009 [0.736, 1.384]	0.953
352	232	105	2.373 [1.540, 3.656]	< 0.001	0.728 [0.438, 1.212]	0.220
376	625	189	2.433 [1.665, 3.554]	< 0.001	0.826 [0.596, 1.145]	0.250
380	930	200	3.805 [2.804, 5.164]	< 0.001	0.784 [0.607, 1.013]	0.063
398	2039	376	2.972 [2.278, 3.877]	< 0.001	0.849 [0.711, 1.014]	0.070
410	904	168	1.603 [1.287, 1.996]	< 0.001	1.069 [0.883, 1.293]	0.494
411	691	214	5.406 [3.891, 7.511]	< 0.001	0.748 [0.561, 0.997]	0.048
428	900	212	3.175 [2.303, 4.379]	< 0.001	0.800 [0.600, 1.069]	0.130
440	910	209	2.376 [1.740, 3.244]	< 0.001	0.736 [0.579, 0.937]	0.013
470	489	48	3.042 [2.039, 4.537]	< 0.001	0.618 [0.459, 0.831]	0.002
499	296	111	3.180 [2.123, 4.764]	< 0.001	0.612 [0.421, 0.889]	0.011
504	1326	391	3.025 [2.333, 3.921]	< 0.001	0.982 [0.769, 1.253]	0.882
528	240	80	2.180 [1.433, 3.316]	< 0.001	0.942 [0.638, 1.390]	0.761
554	269	91	10.908 [5.992, 19.856]	< 0.001	0.750 [0.407, 1.384]	0.354
578	289	100	5.802 [3.428, 9.821]	< 0.001	1.278 [0.815, 2.005]	0.282
616	888	271	2.944 [2.099, 4.130]	< 0.001	1.009 [0.731, 1.393]	0.955
620	790	223	3.448 [2.495, 4.764]	< 0.001	0.757 [0.589, 0.973]	0.030
682	722	292	1.559 [1.131, 2.149]	0.007	0.779 [0.612, 0.993]	0.044
688	964	199	2.642 [2.102, 3.319]	< 0.001	0.887 [0.709, 1.109]	0.291
703	921	237	3.937 [2.827, 5.484]	< 0.001	0.839 [0.642, 1.097]	0.199
704	1388	202	2.221 [1.754, 2.812]	< 0.001	0.951 [0.808, 1.119]	0.544
705	696	214	3.445 [2.540, 4.673]	< 0.001	1.182 [0.881, 1.584]	0.263
710	790	321	2.500 [1.857, 3.367]	< 0.001	0.830 [0.674, 1.021]	0.077
724	1993	502	2.711 [2.262, 3.249]	< 0.001	0.945 [0.800, 1.117]	0.508
752	446	178	2.591 [1.853, 3.623]	< 0.001	0.895 [0.653, 1.228]	0.491
784	1259	212	3.148 [2.217, 4.470]	< 0.001	0.764 [0.582, 1.002]	0.052
792	1299	221	2.007 [1.618, 2.490]	< 0.001	0.851 [0.712, 1.017]	0.076
807	975	201	4.538 [3.426, 6.011]	< 0.001	0.622 [0.502, 0.770]	< 0.001

System ID	N	No. of schools	Utility OR [95% CI]	p	Risk OR [95% CI]	p
840	372	137	5.312 [3.014, 9.360]	< 0.001	0.991 [0.688, 1.427]	0.961
860	1313	210	1.952 [1.593, 2.392]	< 0.001	0.782 [0.673, 0.909]	0.001
9134	395	126	2.741 [1.551, 4.846]	< 0.001	0.720 [0.424, 1.223]	0.222
9642	941	216	3.656 [2.616, 5.110]	< 0.001	0.992 [0.746, 1.320]	0.957
156001	1257	205	1.437 [1.203, 1.715]	< 0.001	0.952 [0.822, 1.102]	0.506

Note. Each education-system model was a binary logistic regression that simultaneously included perceived AI utility, perceived AI risk, AI-related professional learning, age, teaching experience, and highest educational attainment, applied TCHWGT within the system, and adjusted standard errors for clustering at the school level. Factor scores for perceived AI utility and perceived AI risk were the fixed-alignment scores used in the main analysis.

Table S7 Weighted proportions and 95% confidence intervals for eight pedagogical applications of AI among AI users

Item	Application	C1	C2	C3	C4
TT4G37A	supporting assessment	0.242 [0.182, 0.303]	0.179 [0.160, 0.199]	0.350 [0.339, 0.362]	0.407 [0.386, 0.429]
TT4G37B	working with instructional content	0.538 [0.459, 0.617]	0.564 [0.537, 0.592]	0.740 [0.730, 0.751]	0.827 [0.811, 0.843]
TT4G37C	designing lessons and activities	0.444 [0.368, 0.520]	0.524 [0.496, 0.551]	0.725 [0.715, 0.736]	0.834 [0.819, 0.849]
TT4G37D	supporting students with special learning needs	0.279 [0.216, 0.341]	0.211 [0.190, 0.232]	0.432 [0.420, 0.444]	0.571 [0.548, 0.593]
TT4G37E	adjusting material difficulty	0.265 [0.201, 0.329]	0.242 [0.219, 0.265]	0.483 [0.471, 0.495]	0.661 [0.639, 0.683]
TT4G37F	generating feedback and communication texts	0.283 [0.224, 0.343]	0.258 [0.233, 0.282]	0.426 [0.413, 0.438]	0.551 [0.531, 0.572]
TT4G37G	analyzing student engagement and performance	0.290 [0.225, 0.355]	0.206 [0.186, 0.226]	0.372 [0.360, 0.384]	0.415 [0.394, 0.436]
TT4G37H	skill practice in authentic contexts	0.384 [0.311, 0.457]	0.385 [0.359, 0.411]	0.543 [0.530, 0.555]	0.610 [0.588, 0.633]

Note. Cells are weighted proportions [95% CI]. Profile-specific proportions used joint weights equal to posterior class-membership probabilities multiplied by TCHWGT rescaled to sum to 1 within each education system; 95% CIs used Taylor series linearization with school-level clustering. Items use separate valid-response samples and are independent binary indicators, so proportions need not sum to 1. C1 = Indifferent Profile; C2 = Skeptical Profile; C3 = Measured Endorsement Profile; C4 = Risk-Aware Strong Endorsement Profile.

Table S8 Weighted proportions and 95% confidence intervals for five barriers to AI use among non-users

Item	Barrier	C1	C2	C3	C4
TT4G38A	resource and infrastructure constraints	0.370 [0.341, 0.399]	0.378 [0.362, 0.394]	0.440 [0.426, 0.453]	0.571 [0.540, 0.602]
TT4G38B	insufficient knowledge and skills	0.615 [0.586, 0.645]	0.647 [0.631, 0.662]	0.662 [0.650, 0.674]	0.589 [0.558, 0.620]
TT4G38C	reservations about the pedagogical appropriateness of AI	0.833 [0.809, 0.857]	0.673 [0.658, 0.688]	0.366 [0.354, 0.378]	0.218 [0.194, 0.243]
TT4G38D	school-level restrictions on AI use	0.172 [0.149, 0.196]	0.145 [0.134, 0.157]	0.120 [0.111, 0.128]	0.123 [0.099, 0.148]
TT4G38E	feeling overwhelmed by integrating new technologies	0.376 [0.346, 0.406]	0.369 [0.354, 0.385]	0.311 [0.300, 0.322]	0.273 [0.245, 0.302]

Note. Cells are weighted proportions [95% CI]. Profile-specific proportions used joint weights equal to posterior class-membership probabilities multiplied by TCHWGT rescaled to sum to 1 within each education system; 95% CIs used Taylor series linearization with school-level clustering. Items use separate valid-response samples and are independent binary indicators, so proportions need not sum to 1. C1 = Indifferent Profile; C2 = Skeptical Profile; C3 = Measured Endorsement Profile; C4 = Risk-Aware Strong Endorsement Profile.

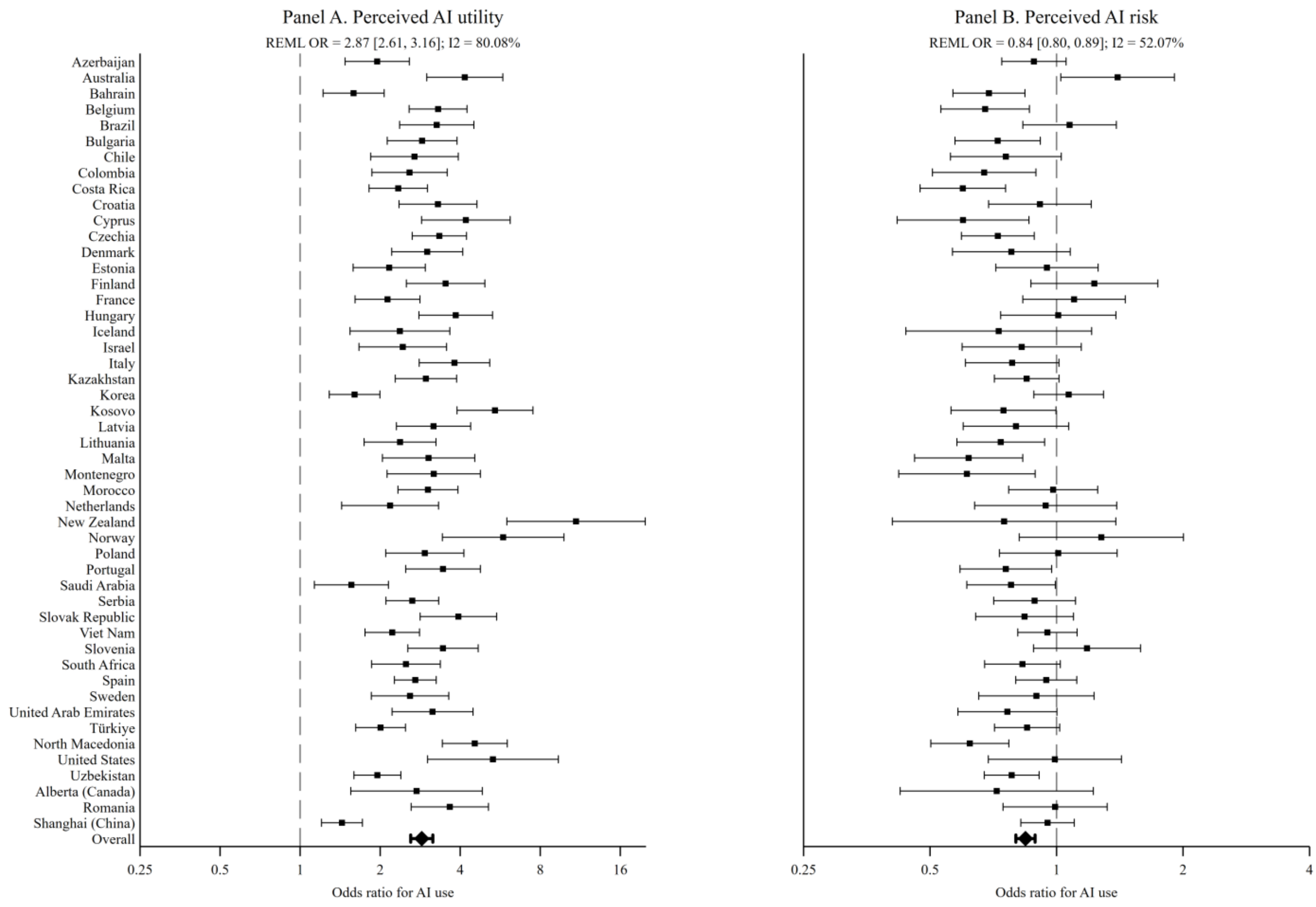

Figure S1 Forest plots of the associations of perceived AI utility and perceived AI risk with AI use across 49 education systems.

Panels A and B show system-specific ORs and 95% CIs for perceived AI utility and perceived AI risk, respectively. Squares and horizontal lines indicate ORs and 95% CIs, diamonds indicate REML random-effects summary estimates, and dashed vertical lines indicate OR = 1. The x-axis is on a logarithmic scale.
